# Performance of gene expression–based single sample predictors for assessment of clinicopathological subgroups and molecular subtypes in cancers: a case comparison study in non-small cell lung cancer

**DOI:** 10.1093/bib/bbz008

**Published:** 2019-02-04

**Authors:** Helena Cirenajwis, Martin Lauss, Maria Planck, Johan Vallon-Christersson, Johan Staaf

**Affiliations:** Division of Oncology and Pathology, Department of Clinical Sciences Lund, Lund University, Medicon Village, Lund, Sweden

**Keywords:** single sample predictor, non-small cell lung cancer, classification, gene pair, molecular subtype, tumor histology

## Abstract

The development of multigene classifiers for cancer prognosis, treatment prediction, molecular subtypes or clinicopathological groups has been a cornerstone in transcriptomic analyses of human malignancies for nearly two decades. However, many reported classifiers are critically limited by different preprocessing needs like normalization and data centering. In response, a new breed of classifiers, single sample predictors (SSPs), has emerged. SSPs classify samples in an N-of-1 fashion, relying on, e.g. gene rules comparing expression values within a sample. To date, several methods have been reported, but there is a lack of head-to-head performance comparison for typical cancer classification problems, representing an unmet methodological need in cancer bioinformatics. To resolve this need, we performed an evaluation of two SSPs [k-top-scoring pair classifier (kTSP) and absolute intrinsic molecular subtyping (AIMS)] for two case examples of different magnitude of difficulty in non-small cell lung cancer: gene expression–based classification of (i) tumor histology and (ii) molecular subtype. Through the analysis of ~2000 lung cancer samples for each case example (*n* = 1918 and *n* = 2106, respectively), we compared the performance of the methods for different sample compositions, training data set sizes, gene expression platforms and gene rule selections. Three main conclusions are drawn from the comparisons: both methods are platform independent, they select largely overlapping gene rules associated with actual underlying tumor biology and, for large training data sets, they behave interchangeably performance-wise. While SSPs like AIMS and kTSP offer new possibilities to move gene expression signatures/predictors closer to a clinical context, they are still importantly limited by the difficultness of the classification problem at hand.

## Introduction

Gene expression profiling by high-throughput methods like microarrays and, more recently, RNA sequencing has been used for nearly two decades in cancer research. Two of the most central aims have been to derive the following: (i) gene expression–based predictors of patient risk of disease relapse (prognostic predictors) or benefit of a specific treatment (treatment response predictors) or (ii) predictors for novel molecular disease subtypes or for established histological subgroups of a disease. A general hope has been that molecular-driven disease stratification could positively affect patient treatment and outcome for cancer patients, ultimately providing a more personalized cancer care. The most well-known example of gene expression–driven disease stratification is likely the now, in clinical routine, established molecular subtypes of breast cancer first reported in early 2000 [1] that stratifies breast cancer into five subtypes.

Over the years, a plethora of different risk predictors as well as molecular subtype predictors have been reported for nearly all human malignancies. However, only a small number has actually been independently validated, and even fewer have made it to a stage of clinical use. Two of several reasons for this lack of clinical translation or reproducibility are likely the predictor development and implementation process itself, as well as lack of sufficiently large and unbiased training cohorts that properly mirror the relevant population-based clinicopathological demographics. In many instances, predictors of risk and molecular subtype proposed for classifying independent cases have been developed in a way that requires gene centering of input data to assure comparable relative gene expression levels across samples. Typical early steps in microarray-based gene expression data processing have included background correction, normalization (different methods for different platforms) and probe filtering (e.g. to remove cross-hybridizing probes). For RNA sequencing data, preprocessing includes alignment, quality filtering and summarization into transcript counts (e.g. Fragments per kilo base per million mapped reads (FPKM)). For both data types, gene/transcript centering across samples is often performed as a 2nd step, which creates gene expression estimates relative to the cohort processed, and no longer absolute in its nature. A classical example of this is the nearest centroid classification (NCC) method. In NCC, each class is characterized by its vector of means (centroid) derived from a training set, to which a similarity score is computed for an unknown sample to identify the class with the best (nearest) match. Many other examples exist with the common theme that classification of independent samples becomes related to both the training set from which the predictor was derived and to the composition of the cohort from which the new sample originates [2–5].

Predictors based on gene rules (gene pairs) have been proposed as a possibility to circumvent the sensitivity of predictors dependent on gene centering and also to be more platform independent [2, 4, 6, 7]. This type of predictor consists of a set of gene decision rules optimized through training versus an endpoint variable (e.g. histological subtype). A decision rule can be explained as ‘if expression of gene A < expression of gene B’, the sample is assigned as class X, otherwise as non-X. Classification may then be performed by a straightforward voting scheme or by other algorithms integrating the results. Predictors of this type may be referred to as (true) single sample predictors (SSPs), as they propose robust classification of new cases truly independent of others. While reports of this predictor type are becoming more frequent [2, 4, 6, 8, 9], to date there has not been a thorough comparison of different SSP predictors for the same classification problem, representing an unmet methodological need in cancer bioinformatics.

In the present study, we aimed to evaluate the classification performance, robustness, platform independence and prediction agreement of two SSPs [absolute intrinsic molecular subtyping (AIMS) [2] and the k-top-scoring pair classifier (kTSP) [6]] for two classification problems of different difficulty, using non-small cell lung cancer (NSCLC) as the context, involving 3213 unique samples from 19 independent studies. AIMS and kTSP represent two different types of SSPs (albeit with a similar final gene rule approach), with AIMS (based on Naïve Bayes statistics) standing out compared to kTSP and similar rank-based methods (e.g. [4, 7]) for its ability to be trained to predict >2 classes. NSCLC is a highly heterogeneous disease at the molecular level, broadly stratified into different histological subtypes [predominantly adenocarcinoma (AC) and squamous cell carcinoma (SqCC)] representing distinct biological entities (albeit with high intergroup heterogeneity).

In this setting, we chose to evaluate the SSPs for two types of classification problems commonly found in transcriptomic studies of cancer: (i) a prediction of histopathological tumor histology (AC or SqCC) and (ii) a prediction of proposed molecular subtypes within a specific tumor histology/subgroup, in our case, exemplified by the terminal respiratory unit (TRU) and non-TRU subtypes [10, 11] within AC. The former case represents a hypothetically easy classification problem based on the biologically distinct AC and SqCC types (including disparate transcriptional patterns, [12–14]). The latter case represents a hypothetically more challenging problem in solid cancers. This is partly due to common intra-tumor heterogeneity with infiltration of different nonmalignant cell populations, coupled with the usually strong relationship of molecular subtypes to generic biological processes such as cell proliferation as well as differences (occasionally subtle) in the tumor microenvironment.

## Materials and methods

### Data sets

**Figure 1:**
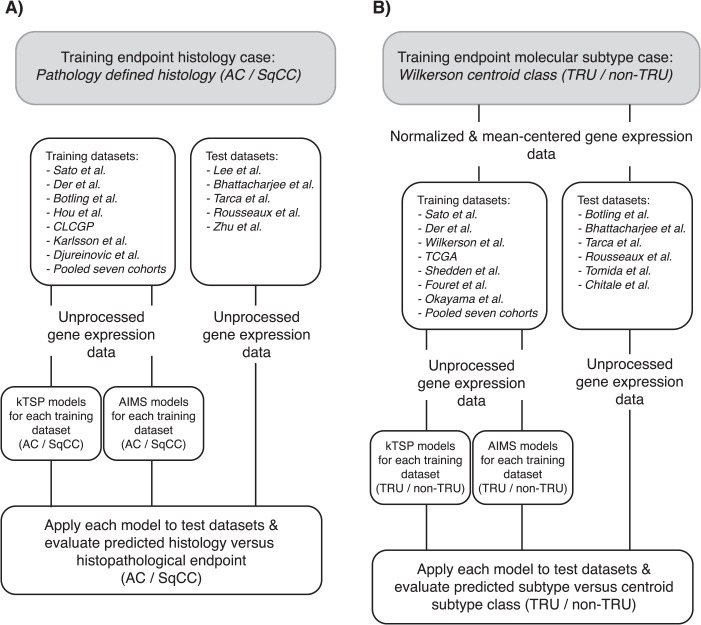
Flowchart of analyses in the study. (A) Histology case arm. (B) Molecular subtype case arm.

Publicly available gene expression data from 19 independent studies (*n* = 3213 unique samples), generated between 2001 and 2016, were collected from public repositories or authors' websites. The cohort collection reflects different ethnicity, with cohorts from both the western world and Asian countries. The individual cohorts varied in the number of samples, gender distribution, tumor stage proportion and histology. Histopathological assessment (e.g. stage and histology) was conducted according to the World Health Organization (WHO) guideline relevant at the time of each respective study, representing a potential source of bias between studies. Overall, cohort characteristics have been summarized in Table 1. For each cohort, detailed clinical information can be found in the original studies. Irrespectively of pronounced variations across cohorts, individual cohorts were assigned in respective entirety as either a ‘training’ or ‘test’ data set. Cohorts were used in two separate case study arms (each comprising both training and test data sets): (i) the histology case study arm (*n* = 1918; 7 training and 5 test data sets) and (ii) the molecular subtype case study arm (*n* = 2106; 7 training and 6 test data sets; Table 1 and Figure 1). Six data sets were shared between both case study arms. To avoid sample overlap between the Shedden *et al.* [15] and Zhu *et al.* [16] cohorts in our analyses, these data sets were intentionally placed in different arms.

**Table 1:** The data sets analyzed in this study

**Data sets**	**Total** (*N*)	**Accession**	**Platform**	**Sex males (%)**	**Stage I (%)**	**Histology AC (%)**	**Molecular TRU (%)**	**Case arm histology**	**Case arm molecular**
Sato *et al.* [29]	263	GSE41271	Illumina	54	50	70	38	Train	Train
Der *et al.* [30]	170	GSE50081	Affymetrix	53	70	75	39	Train	Train
Botling *et al.* [12]	172	GSE37745	Affymetrix	53	64	62	35	Train	Test
Hou *et al.* [31]	72	GSE19188	Affymetrix	65	NA	62	31	Train	
Clinical Lung Cancer Genome Project^a^ [14]	191	CLCGP	Illumina	63	47	51	34	Train	
Djureinovic *et al.* [32]	183	GSE81089	RNAseq	47	58	63	50	Train	
Karlsson *et al.* [33]	99	GSE60644	Illumina	46	90	78	40	Train	
Lee *et al.* [34]	138	GSE8894	Affymetrix	75	NA	46	38	Test	
Bhattacharjee *et al.* [19]	211	GSE83227	Affymetrix	36	40	90	37	Test	Test
Tarca *et al.* [35]	150	GSE43580	Affymetrix	80	50	51	42	Test	Test
Rousseaux *et al.* [21]	146	GSE30219	Affymetrix	84	90	58	34	Test	Test
Zhu *et al.*^b^ [16]	123	GSE14814	Affymetrix	67	54	58	35	Test	
Wilkerson *et al.* [10]	116	GSE26939	Agilent	46	53	100	41		Train
Cancer Genome Atlas Research Network^c^ [11]	230	TCGA LUAD	RNAseq	NA	NA	100	39		Train
Shedden *et al.* [15]	444	Shedden	Affymetrix	50	62	100	38		Train
Fouret *et al.* [36]	103	E_MTAB_923^d^	Affymetrix	16	58	100	42		Train
Okayama *et al.* [37]	226	GSE31210	Affymetrix	46	74	100	43		Train
Tomida *et al.* [38]	117	GSE13213	Agilent	51	68	100	40		Test
Chitale *et al.*^e^ [39]	102	Chitale U133 2plus	Affymetrix	41	69	100	41		Test

^a^CLCGP: The Clinical Lung Cancer Genome Project (http://www.uni-koeln.de/med-fak/clcgp/).

^b^Present data set overlaps with Shedden *et al.* [15] (43 samples).

^c^The Cancer Genome Atlas Network (TCGA).

^d^Data obtained from the ‘ArrayExpress’ database (https://www.ebi.ac.uk/arrayexpress/experiments/E-MTAB-923/).

^e^Samples were divided into two cohorts based on the different Affymetrix platforms, U133A and U133 2plus. Only the latter subset was included in the analysis.

### Preprocessing and centroid classification of gene expression data

In the histology case study arm, non-normalized (raw) gene expression data were used with inclusion of AC and SqCC samples (Figure 1). Two exceptions existed, related to two data sets without publicly deposited raw data (see Supplementary Data). For these data sets, we used the deposited normalized only data. Probes corresponding to RefSeq features were retained and, for duplicated probes, the most varying probe was kept in the data. Each gene was represented by the mean of multiple probes for that specific gene. Common genes across the 12 data sets in this arm were extracted (*n* = 7466 genes). These steps were taken to create a uniform expression matrix with common identifiers across all cohorts for simplicity of the analysis.

In the molecular subtype case study arm, the same procedure was followed but restricted to only AC cases. In addition, to generate training/reference classes for this arm, samples were classified according to the scheme laid out in Wilkerson *et al.* [10] (including normalization and gene centering of data) into one of the three molecular AC subtypes, TRU, proximal-proliferative (PP) and proximal-inflammatory (PI) [11], for each data set separately (see Supplementary Data for details). A two-group class comprising of TRU versus non-TRU cases was generated and used as training/reference class in subsequent analyses (as e.g. kTSP can only be trained for binary outcome). Common genes across the 13 data sets in this arm were extracted (*n* = 7373 genes).

### kTSP single sample predictor

The R package ‘switchBox’ (version 1.16.0) was obtained from the open source software project Bioconductor (http://www.bioconductor.org) [17]. Functions provided by the package were used for training, i.e. building the classifiers based on a histopathological assessment (AC/SqCC) or molecular classification (TRU/non-TRU) in training data sets, and subsequent evaluation of the classifiers in independent test data sets (Table 1). For a detailed description, see Supplementary Data.

### AIMS single sample predictor

For implementation of AIMS [2], we used source scripts available from the project's GitHub repository (https://github.com/meoyo/trainAIMS). A detailed description of the used procedure is found in the Supplementary Data.

### Statistical analyses

All statistical analyses were performed using R [18] (two-sided tests). Kruskal–Wallis tests were performed to compare the classification performance (accuracy) of trained classifiers in test data sets both within and between the two classification methods used (kTSP and AIMS).

Platform independence was examined by stratifying by platform origin of training data sets and merging the outcome (accuracy) of test data sets. Balanced accuracy was defined as (TP/P + TN/N)/2 where TP = true positives, P = positives, TN = true negatives and N = negatives.

## Results

### Study cohorts and baseline classifications

**Figure 2:**
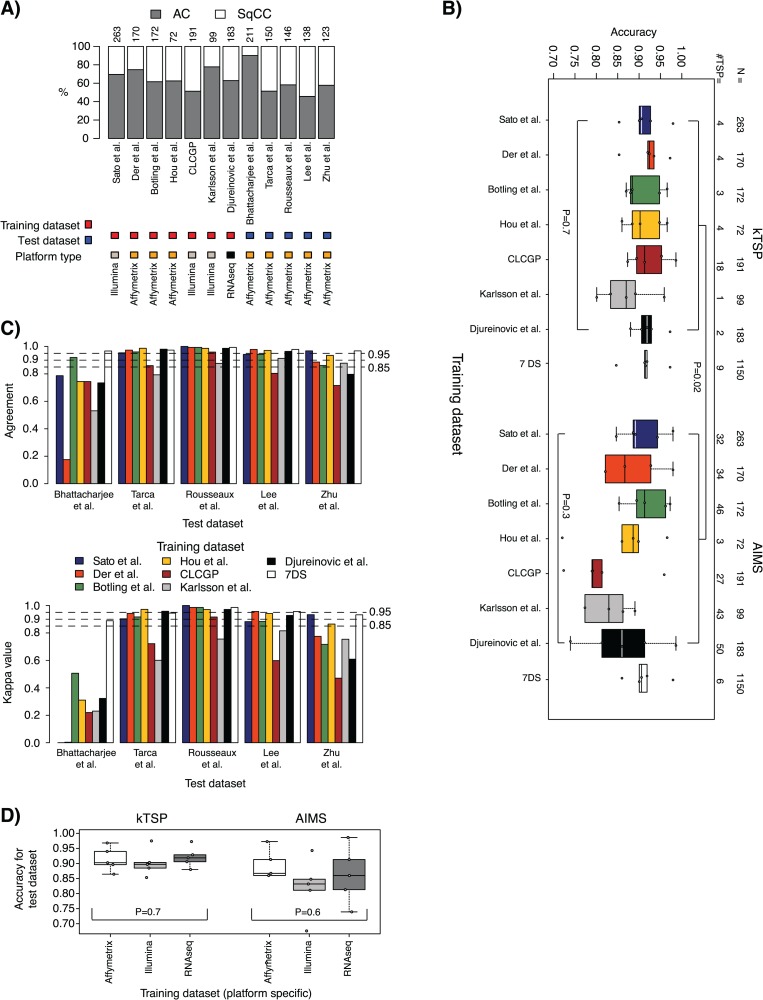
Histology case results. (A) Distribution of tumor type by histopathology (representing the endpoint variable) across training and test data sets. Top axis lists data set sizes. AC, adenocarcinoma; SqCC, squamous cell carcinoma. (B) Accuracy results in test data sets for models trained in the seven individual training data sets plus a pooled training data set of all individual data sets (7DS) and applied to the five test data sets for kTSP and AIMS, respectively. Values below 0.7 are not shown. (C) Top: agreement in SSP predictions between kTSP and AIMS for the models derived from respective training data sets (individual bars, corresponding to, e.g. the classification agreement of a kTSP model versus AIMS model both developed in the Sato *et al.* [29] Illumina cohort applied to the Tarca *et al.* [35] test data set) for each of the five test data sets (group of bars). Agreement is calculated as the proportion of all samples having the same SSP prediction by kTSP and AIMS, irrespective of the histopathological classification. Bottom: corresponding Cohen kappa estimates for the comparison in C. (D) Averaged accuracies for individual test data sets in a platform-wise manner based on training data origin. For classifiers trained on gene expression data run on the same platform, the outcomes (accuracies) across individual test data sets were averaged for kTSP and AIMS, respectively. *P*-values are calculated using Kruskal–Wallis test for the set of groups defined within the hard brackets. For comparisons between two groups each defined by hard brackets, e.g. in panel B, the Mann–Whitney test was used.

**Figure 3:**
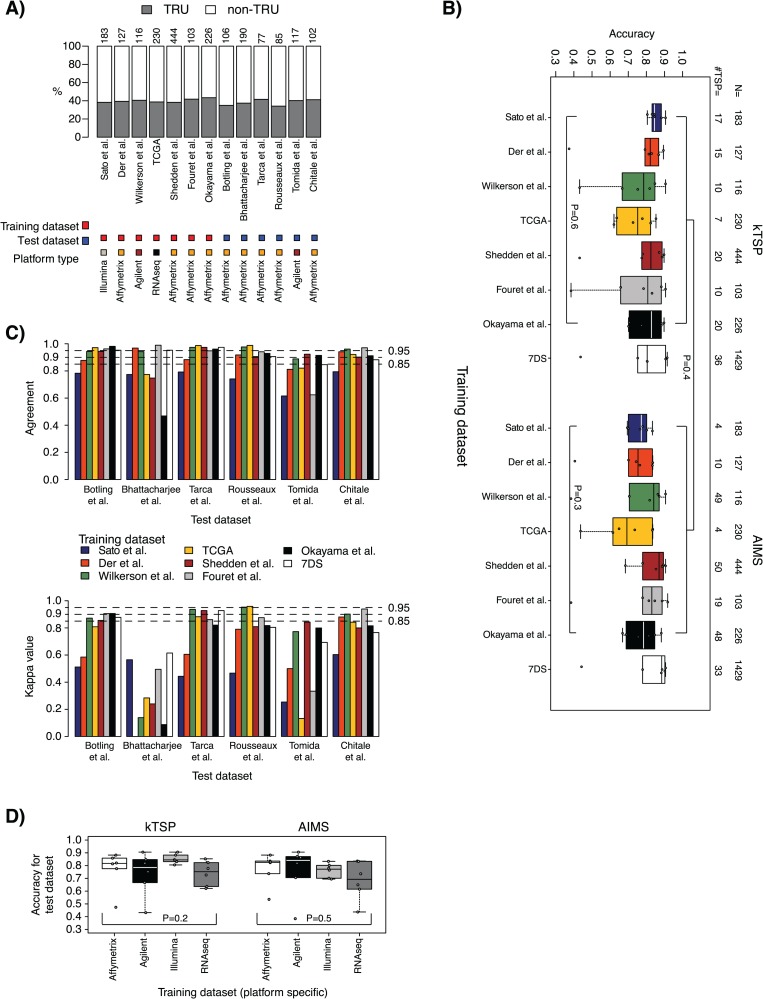
Molecular subtype case results. (A) Distribution of molecular subtype (representing the endpoint variable) across training and test data sets based on original centroid classification according to Wilkerson *et al.* [10]. Top axis lists data set sizes. (B) Accuracy results in test data sets for models trained in the seven individual training data sets plus a pooled training data set of all individual data sets (7DS) and applied to the six test data sets for kTSP and AIMS, respectively. (C) Top: agreement in SSP predictions between kTSP and AIMS for the models derived from respective training data sets (individual bars, corresponding to, e.g. the classification agreement of a kTSP model versus AIMS model both developed in the Sato *et al.* [29] Illumina cohort applied to the Tarca *et al.* [35] test data set) for each of the six test data sets (group of bars). Agreement is calculated as the proportion of all samples having the same SSP prediction by kTSP and AIMS, irrespective of the basic centroid-based molecular subtype classification. Bottom: corresponding Cohen kappa estimates for the comparison in C. (D) Averaged accuracies for individual test data sets in a platform-wise manner based on training data origin. For classifiers trained on gene expression data run on the same platform, the outcomes (accuracies) across individual test data sets were averaged for kTSP and AIMS, respectively. *P*-values are calculated using Kruskal–Wallis test for the set of groups defined within the hard brackets. For comparisons between two groups each defined by hard brackets, e.g. in panel B, the Mann–Whitney test was used.

In the present study, gene expression data from 19 independent studies (Table 1), performed on different gene expression platforms, were divided into ‘training’ and ‘test’ data sets as whole entities in two separate case study arms: a histology case arm based on standard WHO histopathological assessment (AC or SqCC) and a molecular subtype case arm based on previously reported AC molecular subtypes in a binary constellation, i.e. TRU versus non-TRU [3, 10, 11] (Figure 1 and Table 1). In the histology case study arm, both AC and SqCC samples were included. The proportion of AC samples varied between 46–90% across the 12 included data sets, which also varied in size from 72 to 263 samples (Figure 2A and Table 1). In the molecular subtype case study arm, only AC samples were considered. The fraction of TRU classified samples according to the originally reported classifier [10, 11] varied between 34–43% across the 13 data sets included, which also varied in size from 77 to 444 samples (Figure 3A and Table 1). Both experimental arms were subjected to two SSPs, kTSP and AIMS, using all available gene expression data, to elucidate their classification performance on pre-classified data of clinicopathological or molecular character representing different degrees of classification difficulty. For both AIMS and kTSP, we used unprocessed raw expression data, without any within sample or across sample normalization or gene centering (see Supplementary Data). Preprocessed gene expression data was only used to generate molecular subtype predictions in the corresponding case study arm.

### Predicting lung cancer histology by single sample gene expression classifiers

In the histology case study arm, the classification accuracy of the seven kTSP classifiers trained from individual training data sets ranged from 0.80 to 0.99 [median accuracy = 0.91; interquartile range (IQR) = 0.07] across the five test data sets (Figure 2B; Supplementary Figure 1A). Corresponding balanced accuracies ranged from 0.50 to 0.98 (median = 0.89; IQR = 0.08; Supplementary Figure 1B). The optimized number of gene decision rules behind the classifiers, also called top-scoring pairs, ranged from 1 to 18 (Figure 2B). The trained kTSP classifiers performed equally well on assigning patients in test data sets into histological subtypes [*P* = 0.7 (accuracy) or *P* = 0.8 (balanced accuracy not shown), Kruskal–Wallis test; Figure 2B].

The classification accuracy of the corresponding seven AIMS classifiers from the individual training data sets ranged from 0.11 to 0.99 (median = 0.87; IQR = 0.11; Figure 2B; Supplementary Figure 2A). Balanced accuracies ranged from 0.51 to 0.98 (median = 0.88; IQR = 0.09; Supplementary Figure 2B). The number of optimized gene rules behind the different AIMS classifiers ranged from 3 to 50 (Figure 2B). There was no difference in classification performance between the trained AIMS classifiers [*P* = 0.3 (accuracy) or *P* = 0.4 (balanced accuracy, not shown), Kruskal–Wallis test; Figure 2B].

Notably, one test data set, Bhattacharjee *et al.* [19], revealed poorer performance for both classifier types for accuracy (AIMS) or balanced accuracy (kTSP and AIMS) for several different training data sets (Supplementary Figure 1B and 2). This data set was analyzed by the old HG_U95Av2 Affymetrix platform [19] and not the much more commonly used HG_U133 platform, which could potentially affect the results.

When comparing the overall result obtained by kTSP and AIMS using the individual seven training data sets, a significant difference was observed in accuracy (*P* = 0.02) favoring kTSP, but not in balanced accuracy (*P* = 0.5, not shown), (Mann–Whitney–Wilcoxon test; Figure 2B). However, when increasing the size of the training data set to 1150 cases by merging all 7 individual training data sets, no difference in classification performance between the two methods was found (*P* = 0.8*,* accuracy; *P* = 0.8*,* balanced accuracy; Mann–Whitney–Wilcoxon test). The merged kTSP classifier ranged in classification accuracy from 0.85 to 0.97 (median = 0.91; IQR = 0.006) and balanced accuracy from 0.84 to 0.97 (median = 0.91; IQR = 0.02) across the five test data sets (Figure 2B; Supplementary Figure 1), whereas the merged AIMS classifier ranged from 0.86 to 0.98 (accuracy and balanced accuracy: median = 0.91; IQR = 0.02; Figure 2B; Supplementary Figure 2).

Next we investigated whether kTSP and AIMS predicted samples similarly (irrespectively of underlying histopathology). The level of agreement (% of samples predicted similarly) was generally very high (>85%) for classifiers derived from individual training data sets, except in the Bhattacharjee *et al.* [19] test data set (Figure 2C). For the merged training data set (7DS), agreement was strikingly high (>95%) between the two methods across the five test data sets. Similar findings were found when computing kappa values, with classifiers from the merged training data set having extraordinarily high kappa values (Figure 2C).

To investigate gene expression platform dependency, the outcomes (accuracy or balanced accuracy metrics) for each trained classifier were merged (averaged) in a platform-wise manner based on the training data origin, for each classifier method separately. Distribution of the seven training data sets into three platform origins was as follows: Illumina, *n* = 3 data sets; Affymetrix, *n* = 3 data sets; RNAseq, *n* = 1 data set (Table 1). Both kTSP and AIMS showed platform independency for accuracy metrics (*P* = 0.7 or *P* = 0.6, respectively) and balanced accuracy (*P* = 0.8 or *P* = 0.4, respectively) (Mann–Whitney–Wilcoxon test; Figure 2D; Supplementary Figure 3). These results imply that both methods can provide classifiers trained on gene expression data derived from any platform to predict test data of an unrelated platform.

### Predicting molecular subtypes in lung adenocarcinoma using single sample gene expression classifiers

In the molecular subtype case study arm, the classification accuracy of the seven kTSP classifiers trained from individual training data sets ranged from 0.37 to 0.91 (median = 0.82; IQR = 0.15) across the six test data sets (Figure 3B; Supplementary Figure 4A). Corresponding balanced accuracies ranged from 0.50 to 0.90 (median = 0.80; IQR = 0.15; Supplementary Figure 4B). The number of gene decision rules behind the classifiers ranged from 7 to 20 (Figure 3B). The trained kTSP classifiers performed equally well on assigning test data sets into molecular subtypes for both accuracy (*P* = 0.6) and balanced accuracy (*P* = 0.4, data not shown) (Kruskal–Wallis test; Figure 3B).

Corresponding classification accuracy of the seven trained AIMS classifiers ranged from 0.38 to 0.92 (median = 0.79; IQR = 0.15) across test data sets (Figure 3B; Supplementary Figure 5A). Balanced accuracies ranged from 0.51 to 0.91 (median = 0.76; IQR = 0.20; Supplementary Figure 5B). The number of gene rules behind the classifiers ranged from 4 to 50 (Figure 3B). There was no difference in classification performance between the trained AIMS classifiers for accuracy (*P* = 0.3) or balanced accuracy (*P* = 0.2, data not shown) (Kruskal–Wallis test; Figure 3B). Again, several of the trained molecular subtype classifiers revealed a lower classification performance when predicting the Bhattacharjee *et al.* [19] data set (Supplementary Figure 4 and 5).

Comparison of the accuracy metrics obtained by the two classification methods using the seven training data sets revealed no significant difference between the methods for accuracy (*P* = 0.4) or balanced accuracy (*P* = 0.2, data not shown) (Mann–Whitney–Wilcoxon test; Figure 3B). Again, when increasing the size of the training data set to 1429 cases by merging all individual training data sets, no difference in classification performance across the methods was found for accuracy (*P* = 0.7) or balanced accuracy (*P* = 0.7) (Mann–Whitney–Wilcoxon test). The merged kTSP classifier ranged in classification accuracy from 0.44 to 0.91 (median = 0.80; IQR = 0.12) and from 0.55 to 0.93 for balanced accuracy (median = 0.81; IQR = 0.10) across the six test data sets (Figure 3B; Supplementary Figure 4), whereas the merged AIMS classifier varied between 0.44 to 0.91 in accuracy (median = 0.88; IQR = 0.09) and from 0.55 to 0.92 for balanced accuracy (median = 0.85; IQR = 0.14; Figure 3B; Supplementary Figure 5). The agreement in predicted molecular subtype status between kTSP and AIMS (irrespective of centroid status) was generally high in most test data sets, again with the merged training data set showing stable results in all test data sets (Figure 3C).

kTSP and AIMS both showed gene expression platform independency when comparing accuracy (*P* = 0.2 or *P* = 0.5, respectively) or balanced accuracy metrics (*P* = 0.09 or *P* = 0.3, respectively) (Mann–Whitney–Wilcoxon test; Figure 3D; Supplementary Figure 6). In this comparison, distribution of the seven training data sets into four platform origins was as follows: Illumina, *n* = 1 data set; Affymetrix, *n* = 4 data sets; Agilent, *n* = 1 data set; RNAseq, *n* = 1 data set (Table 1). Here, only the Affymetrix platform included averaged data points.

### Comparison of derived gene pairs and rules between AIMS and kTSP—histology case

**Figure 4:**
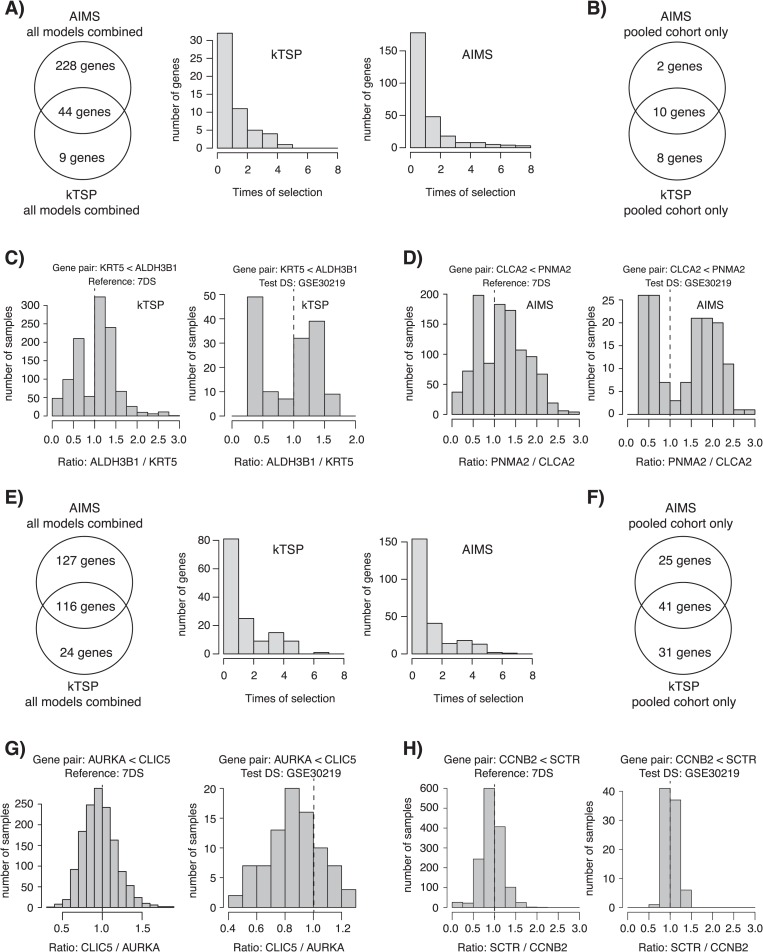
Characteristics of optimized SSP gene pairs for the histology and molecular subtype cases. (A) Left: Venn diagram of overlap between kTSP and AIMS for all genes identified in any of the created models for the histology case arm. Right: number of times a gene was identified in any of the models created in the histology case arm for kTSP and AIMS, respectively. (B) Specific gene overlap of SSP genes between AIMS and kTSP for the SSP models created from the pooled training data set (7DS) in the histology case arm. (C) Left: gene pair expression ratio for all samples in the pooled training data set (7DS) for one kTSP SSP gene pair (*KRT5*<*ALDH3B1*) for the histology model. Right: gene pair expression ratio for the same gene pair in the independent test set GSE30219. In both instances, a bimodal distribution is seen. (D) Similar data as in (C), but now for AIMS for the *CLCA2*<*PNMA2* gene rule. Again, bimodal distributions are seen. (E) Left: Venn diagram of overlap between kTSP and AIMS for all genes identified in any of the created models for the molecular subtype case arm. Right: number of times a gene was identified in any of the models created in the molecular subtype case arm for kTSP and AIMS, respectively. (F) Specific gene overlap of SSP genes between AIMS and kTSP for the SSP models created from the pooled training data set (7DS) in the molecular subtype case arm. (G) Left: gene pair expression ratio for all samples in the pooled training data set (7DS) for one kTSP SSP gene pair (*AURKA*<*CLIC5*) for the molecular subtype model. Right: gene pair expression ratio for the same pair in the independent test set GSE30219. In both instances, a unimodal distribution is seen. (H) Similar data as in (G), but now for AIMS for the *CCNB2*<*SCTR* gene rule. Again, unimodal distributions are seen.

When comparing the individual genes included in the derived gene decision rules from the AIMS and kTSP methods for the histology case example, we found that all rules used in the AIMS classifiers came from 272 unique genes, whereas 53 unique genes were used to build all rules in the kTSP classifiers (Figure 4A). The number of genes overlapping between the kTSP and AIMS methods was 44 in total, with 83% (44/53) of the kTSP genes and 16% (44/272) of the AIMS genes overlapping (Figure 4A). Across all developed models for each method, only a small number of genes were consistently selected in all models (Figure 4A). This may not be surprising given the redundancy and expression correlation of many genes within a specific biological pathway allowing different genes with the same biological function to be selected.

To focus specifically on one model, we chose the high-performing merged training cohort model (Figure 2, 7DS). For this model, 10 (*KRT5*, *DSG3*, *DSC3*, *CLCA2*, *PKP1*, *ALDH3B1*, *PLEKHA6*, *FMO5*, *PNMA2* and *RORC*) of 12 (83%) selected AIMS genes overlapped with the kTSP genes (vice versa, 10/18, 56%; Figure 4B). A total of 8 of the 10 genes were also significantly overlapping with a previously reported expression signature for histological classification of NSCLCs [20] (*P* = 1e-19). A functional enrichment analysis (http://pantherdb.org) for the 10 genes identified cornification as an enriched main biological process with keratinization as subcategory (Supplementary Table 1). This finding is in perfect agreement with KRT5 being a diagnostic immunohistochemistry (IHC) marker of SqCC and that keratinization is a hallmark of squamous cell types.

**Figure 5:**
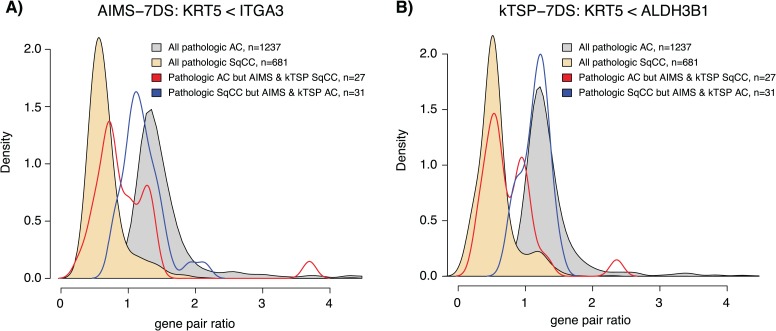
Characteristics of misclassified tumors in the histology case study arm. Gene pair rules were obtained from respective 7DS training data set for AIMS and kTSP. (A) Gene pair ratios for the AIMS KRT5<ITGA3 gene rule stratified by tumor histology and SSP class. (B) Gene pair ratios for the kTSP KRT5<ALDH3B1 gene rule stratified by tumor histology and SSP class. Density plots were created using the density() function in R.

Next we turned to the expression pattern of the selected gene pairs, specifically the effect size (difference in expression) within a gene pair for the selected 7DS models. For each model and gene pair (e.g. gene A > gene B), we formed the ratio of gene A/gene B using the raw unprocessed gene expression estimates for each sample and plotted these values for each sample in the 7DS training cohort and in an independent test set (GSE30219 [21]). Strikingly, all gene pairs formed an apparent bimodal effect size distribution in both the training and independent test data set for both kTSP and AIMS (Figure 4C and D and Supplementary Figure 7 and 8 for all data). The bimodal distribution was present also when stratifying patients by technical gene expression platform (Supplementary Figure 11). The identified gene pair rules, especially the KRT5<ITGA3 (AIMS) and the KRT5<ALDH3B1 (kTSP) rules, may also be used to investigate cases that are misclassified by both SSPs compared to the histopathological endpoint. Interestingly, a large proportion of such cases, especially tumors that were classified as AC by both SSPs but SqCC by histopathology, appears to have a gene pair ratio expression in line with the overall histopathological population of tumors with the same class as predicted by the SSPs (Figure 5). This observation suggests that such cases may actually be of the wrong endpoint or possibly of mixed histological subtypes like adenosquamous NSCLC. Taken together, these results support the biological distinction between the endpoint classes (histology) and the ‘simplicity’ of the classification problem and explain the high performance of the different classifier models.

### Comparison of derived gene pairs and rules between AIMS and kTSP—molecular subtype case

In the molecular subtype case, 243 unique genes were found across all AIMS models, whereas 140 unique genes were used to build all rules in the kTSP classifiers (Figure 4E). The number of genes overlapping was 116 in total, with 83% (116/140) of the kTSP genes and 48% (116/243) of the AIMS genes overlapping (Figure 4E). Similarly as for the previous case example, only a small number of genes were consistently selected in all models for both methods across all developed models (Figure 4E). To focus specifically on one model, we again chose the high-performing merged training cohort model (Figure 3, 7DS). For this model, 41 genes overlapped between both methods (Figure 4F), strongly enriched for biological processes associated with cell proliferation, cell division and mitotic spindle organization, e.g. *CCNA2*, *CCNB1*, *CDC20* and *AURKA* consistent with reports of expression of proliferation-related genes being a main divider between TRU and non-TRU cases [3, 22] (Supplementary Table 1). Moreover, 27 out of the 41 genes were also overlapping with the centroids originally published by Wilkerson *et al.* [10] and used in this study for classification of AC into intrinsic molecular subtypes (*P =* 1.55e-25).

A similarly performed effect size analysis using the raw unprocessed gene expression estimates for each sample revealed a different overall pattern for selected gene pairs for both methods (7DS training, test set GSE30219 [21]). Instead of a bimodal distribution, gene pair effect distributions appeared more unimodal in both training and test data sets (Figure 4G and H and Supplementary Figure 9 and 10 for all data). This pattern remained also when stratifying samples for technical platforms (Supplementary Figure 11). Together, these observations are in line with our previous studies on the Wilkerson subtypes and that proliferation when measured by gene expression represents a unimodal distribution of values [3]. These results highlight the difficulty of this classification problem in that the subtypes are not equally distinct, providing a background to the lower classifier performance observed (Figure 3).

## Discussion

In the present study, we have investigated an unresolved methodological question in cancer bioinformatics, related to the usage of (true) gene expression–based SSPs. Based on two lung cancer classification scenarios, selected to represent different levels of classification difficulty, we assess two SSP methods (kTSP and AIMS) for performance, robustness, platform independence and agreement.

The 1st classification scenario, a prediction of lung cancer AC or SqCC histology, represents a hypothetically easy classification problem, due to classes with separate and distinctive biology (and hence different active transcriptional programs). For this case example, we observed high accuracy (median ~0.87–0.92) in predictions across five test data sets for both SSP methods versus the endpoint variable (tumor type by histopathology) for nearly all developed models (Figure 2). These accuracies are in line with what has been reported for non-SSP–based machine learning/prediction methods in smaller studies [20]. Noteworthy, in this case example, both training and test data sets differ greatly in histological subtype proportions, cohort size and also technical platforms used to generate the expression data. Despite the high agreement, it should be noted that histopathological assessment of histology for both training and test sets was conducted according to the WHO guideline relevant at the time of the original studies, representing a source of variation/discrepancy. It may be expected that if histology predictions had been reevaluated using the current WHO 2015 guidelines through central histopathological review, a proportion of cases would have been reclassified (e.g. in [23] ~5% of cases were reclassified between WHO 2004 and 2015 guidelines). This may account for some of the discrepancy between histopathological and gene expression–based predictions.

While it appears feasible to derive accurate SSPs from small cohorts for this case example (involving distinct biological entities), we do note a greater stability (less variation) in accuracy for test data sets when combining cohorts into larger meta-sets for training for both SSP methods (Figure 2B), despite differences in cohort size, technical platform and endpoint proportions. This implies that the distinct transcriptional programs in the SSP training override technical platform differences. One support for this claim is the technical platform insensitivity of the gene ratio distributions calculated for each method in both case study arms (Supplementary Figure 11). This platform robustness is further illustrated by the performance of the pooled models but also the high performance of models derived from, e.g. unprocessed RNAseq data, which predicts histology in microarray cohorts generated on different commercial platforms with good accuracy (Figures 2C and 3C). Notably, pooling of gene expression data sets has proven problematic since the early days of the technique for more conventional types of analysis (involving analysis of relative expression across samples). Besides providing an apparent option for pooling cohorts for training, SSP models may also provide a more transparent option to also pool validation cohorts to provide greater power in, e.g. outcome analyses, as no consideration of validation cohort composition is needed in the actual prediction. Hence, SSP methods, relying on relative expression within samples only, may thus have a competitive edge for deriving stable classifiers. In addition, when pooling data sets for training, we observe that the two SSP methods display extraordinary agreement in their individual sample classification (Figure 2C). Interestingly, there is a large overlap of genes selected for the gene rules between the two different methods, especially considering that >7000 genes were available for training. The overlap becomes even more striking considering the biology of the selected genes, exemplified by the models derived from the pooled training data set (combining seven individual data sets). In these models, overlapping model genes are highly enriched for biology related to keratinization, a hallmark of SqCC, and also includes a diagnostic marker gene (cytokeratin 5, *KRT5*) used in clinical practice today for IHC-driven classification of NSCLC histology. Moreover, our deepened analysis of the selected gene pairs for this case example reveals that the type of gene pairs selected displays expression ratios with an apparent bimodal distribution independent of technical platform. The latter greatly facilitates prediction. Taken together, for a gene expression classification problem of similar degree of difficulty, it appears that if a sufficiently large training set exists, the two tested methods perform very robust, are platform independent, identify actual biology and are interchangeable.

The 2nd case example was chosen to be more challenging for predictor development, namely a prediction of (reported) gene expression–based molecular subtypes of AC [10, 11]. In contrast to the 1st case example, we now operate within a single histological subgroup of lung cancer (AC). Although transcriptional patterns of lung ACs have been studied for a long time (and found to be heterogeneous), it is still debatable whether specific, robust gene expression subtypes exist, and if so which they are (see e.g. [22] for a metastudy-like comparison of different reported subtypes). In this case example, we encounter multiple difficulties in predictor development, all of which likely contribute to and explains the generally lower performance of SSP methods versus the endpoint variable. Firstly, the technical training endpoint class (TRU/non-TRU) itself is problematic as it is defined from gene-centered data by NCC, a classification approach shown by us and others to be sensitive to cohort composition [2, 3, 24–27], if special considerations like external/internal centering cohorts are not taken. Figure 3A illustrates this problem by showing the highly similar endpoint class proportions across data sets, something that would not be expected given the difference in clinicopathological characteristics of the individual cohorts (Table 1). Thus, the endpoint in the training data sets is already from the start likely skewed/biased. Secondly, the proposed molecular subtypes are not as distinct biological entities compared to tumor histology types (see e.g. [3, 22] for an in-depth analysis of the TRU/non-TRU subtypes). Typically, classification of similar molecular subtypes, or for that matter other subgroups defined from unsupervised analyses or a priori defined patient groups, often involves making cut-points in gene sets related to biological processes that are usually not bimodal in expression. Examples of such processes include cell proliferation or expression of immune-related genes (related to e.g. the degree of immune cell infiltration in the tumor microenvironment) [3]. Indeed, in our gene rule effect size analysis in this case example, we observed exactly this phenomenon. Thus, we believe this case example represents an important and frequent scenario that researchers often encounter when attempting to transfer findings from, e.g. unsupervised analysis into a predictor for supervised validation. Taken together, it thus needs to be appreciated that a predictor cannot be better than the underlying uncertainty in the training endpoint and that this issue should be properly investigated prior to choosing the validation approach.

Despite the challenges connected to this case example, we do note median accuracies of the different developed models between 0.76–0.88, although with a more pronounced variation compared to the histological case example (Figure 3B). However, it needs to be stressed that the underlying ‘biological’ truth in the classification (prediction or endpoint variable) is difficult to assess due to the nature of the problem at hand. Interestingly, when comparing our median accuracies for the TRU/non-TRU example versus results reported for the AIMS method in a similar prediction setting in breast cancer (PAM50 subtypes, original AIMS study [2]), they are in the same range (~76% agreement). Again, it does appear reasonable to develop predictors in/or across combined data sets with different technical platforms with high agreement in sample classification between methods and overlapping genes in selected gene rules (Figure 3). Again, this indicates a generally similar concept of interchangeability between the methods provided a sufficient number of training samples.

In addition to the presented results, one can pose multiple additional questions about the impact of different low-level data analysis steps versus SSP performance. We analyzed three such questions through exploratory analyses. Firstly, one question not originally addressed in our evaluation is whether different number of input genes affects performance, i.e. feature selection prior to model optimization. To investigate this, we performed a simple exploratory analysis for the 2nd case example, repeating the entire model training scheme, but restricting the number of genes for training to only the overlapping genes present in the original subtype centroids (*n* = 383 of in total 506 original centroid genes). When comparing accuracy metrics obtained from models from the seven training data sets across the test data sets, we did not see any clear trend favoring the original or restricted (directed) gene approach (Supplementary Figure 12). Secondly, we tested the impact of standard normalization (as described in Supplementary Data) versus no normalization by training kTSP SSPs on normalized data instead of non-normalized data, followed by a prediction of non-normalized validation sets. We observed similar accuracy metrics as the original SSPs for both the histology and molecular subtype case study arms (Supplementary Figure 13), indicating that conventional normalization procedures (without gene centering) do not substantially influence SSP performance on a group level. Thirdly, we investigated whether higher centroid correlation resulted in higher prediction accuracies for AIMS and kTSP in validation samples in the molecular subtype case arm. A higher centroid correlation implies a more subtype-like sample, and intuitively such cases could be thought to be more easily (correctly) predicted. However, raising the centroid correlation cut-off from the original highest value up to 0.4 changed the overall prediction accuracy modestly, while simultaneously causing up to ~50% of the validation cases to be unclassified (i.e. without a subtype class due to too low correlation; Supplementary Figure 14). While a trend of higher prediction accuracy is clearly seen with more stringent centroid correlation cut-off, the results indicate that SSPs can handle also cases with lower similarity (correlation) to a subtype centroid.

In summary, we demonstrate that the two tested SSP methods performed as expected generally better for the hypothesized simpler prediction problem. Importantly, we show that the methods displayed good to very good platform independency, they appear largely interchangeable for larger training cohorts, they do appear to select gene rules with relevant biology and that larger training sets (irrespective of expression platform) appear to be beneficial for robustness. Clearly, the overall performance of these predictors appears mostly limited to the difficultness of the classification problem at hand and likely less to the type of model (as also noted previously by MAQC-II for other classifier types [28]). An interesting future development in the field would be rule-based SSPs accounting for effect size between gene pairs. Our study shows that one can find combinations of single small/large data set for training and test that would allow either underestimation or overestimation of a developed classifier. Clearly, assessing traits of molecular predictors appears more robust when using a combined training set and multi-cohort evaluation as best practice. Irrespectively, we argue that development of SSPs for promising gene signatures represents an important way forward to bring gene expression–based predictions closer to clinical use.

## Supplementary Material

Supp_bbz008

## Notes

Key Points

- SSPs represent an alternative to reduce problems associated with classical predictor algorithms in a gene expression–based prediction of various endpoints, including molecular subtypes and risk predictors.
- SSPs benefit of large training data sets and appear to be platform independent.
- For large training data sets, available prediction methods appear interchangeable regarding results for both simpler and more difficult classification problems.
- SSPs are no remedy for difficult prediction problems with less distinct endpoints to predict.

## Notes

**Helena Cirenajwis**, PhD, is a postdoc at Lund University, Sweden. Her research is focused on developing algorithms for prediction of molecular subtypes in lung cancer.

## Notes

**Martin Lauss**, PhD, is an assistant researcher at Lund University, Sweden. His research is mainly focused on the genomics of malignant melanoma.

## Notes

**Maria Planck**, MD, PhD, is an associate professor and senior oncologist at Lund University, Sweden. Her research involves using lung cancer genomics as tools for improving the clinical management of lung cancer.

## Notes

**Johan Vallon-Christersson**, PhD, is an assistant professor at Lund University, Sweden. His research is mainly focused on gene expression profiling of breast cancer.

## Notes

**Johan Staaf**, PhD, is an associate professor of experimental oncology at Lund University, Sweden. His research is focused on translational breast and lung cancer genomics.
