# Supplementary material for: Performance of gene expression–based single sample predictors for assessment of clinicopathological subgroups and molecular subtypes in cancers: a case comparison study in non-small cell lung cancer
Source: Brief Bioinform. 2019 Feb 4;21(2):729–40. doi: 10.1093/bib/bbz008 (PMC7299291; doi:10.1093/bib/bbz008)
Supplement: Supp_bbz008 [file supp_bbz008.zip › SupplementaryFigures.pdf]

SFigure 1

A)

Accuracy for kTSP models

Training dataset

Test dataset	Sato et al. (N=263, #TSP=4)	Der et al. (N=170, #TSP=4)	Botling et al. (N=172, #TSP=3)	Hou et al. (N=72, #TSP=4)	CLCGP (N=191, #TSP=18)	Karlsson et al. (N=99, #TSP=1)	Djureinovic et al. (N=183, #TSP=2)	7DS (N=1150, #TSP=9)
Bhattacharjee et al.	0.9	0.92	0.95	0.95	0.95	0.8	0.93	0.91
Tarca et al.	0.85	0.85	0.88	0.86	0.87	0.83	0.88	0.85
Rousseaux et al.	0.98	0.97	0.97	0.97	0.99	0.96	0.97	0.97
Lee et al.	0.91	0.92	0.88	0.88	0.91	0.89	0.91	0.91
Zhu et al.	0.93	0.93	0.87	0.9	0.89	0.87	0.92	0.92

B)

Balanced accuracy for kTSP models

Training dataset

Test dataset	Sato et al. (N=263, #TSP=4)	Der et al. (N=170, #TSP=4)	Botling et al. (N=172, #TSP=3)	Hou et al. (N=72, #TSP=4)	CLCGP (N=191, #TSP=18)	Karlsson et al. (N=99, #TSP=1)	Djureinovic et al. (N=183, #TSP=2)	7DS (N=1150, #TSP=9)
Bhattacharjee et al.	0.5	0.94	0.74	0.84	0.76	0.85	0.83	0.93
Tarca et al.	0.85	0.85	0.88	0.86	0.87	0.83	0.88	0.84
Rousseaux et al.	0.98	0.97	0.96	0.96	0.98	0.96	0.97	0.97
Lee et al.	0.91	0.92	0.89	0.89	0.91	0.89	0.91	0.91
Zhu et al.	0.92	0.94	0.85	0.89	0.89	0.88	0.91	0.91

Supplementary Figure 1. Accuracy metrics for kTSP models using histopathological assessment (AC/SqCC) as endpoint variable. Classification performance of the kTSP algorithm trained on seven individual training datasets plus a pooled training dataset (7DS) and applied to five test datasets: (**A**) Accuracy. (**B**) Balanced accuracy.

SFigure 2

A)

Accuracy for AIMS models

Training dataset

Test dataset	Sato et al. (N=263, #TSP=32)	Der et al. (N=170, #TSP=34)	Botling et al. (N=172, #TSP=46)	Hou et al. (N=72, #TSP=3)	CLCGP (N=191, #TSP=27)	Karlsson et al. (N=99, #TSP=43)	Djureinovic et al. (N=183, #TSP=50)	7DS (N=1150, #TSP=6)
Bhattacharjee et al.	0.89	0.11	0.96	0.72	0.79	0.35	0.74	0.9
Tarca et al.	0.85	0.87	0.85	0.86	0.81	0.77	0.86	0.86
Rousseaux et al.	0.98	0.98	0.97	0.97	0.96	0.89	0.99	0.98
Lee et al.	0.89	0.93	0.91	0.9	0.79	0.86	0.91	0.91
Zhu et al.	0.94	0.82	0.89	0.89	0.72	0.83	0.81	0.92

B)

Balanced accuracy for AIMS models

Training dataset

Test dataset	Sato et al. (N=263, #TSP=32)	Der et al. (N=170, #TSP=34)	Botling et al. (N=172, #TSP=46)	Hou et al. (N=72, #TSP=3)	CLCGP (N=191, #TSP=27)	Karlsson et al. (N=99, #TSP=43)	Djureinovic et al. (N=183, #TSP=50)	7DS (N=1150, #TSP=6)
Bhattacharjee et al.	0.94	0.51	0.96	0.76	0.88	0.64	0.86	0.92
Tarca et al.	0.84	0.86	0.85	0.86	0.81	0.78	0.86	0.86
Rousseaux et al.	0.98	0.98	0.97	0.96	0.96	0.91	0.98	0.98
Lee et al.	0.89	0.93	0.91	0.9	0.77	0.85	0.91	0.9
Zhu et al.	0.94	0.84	0.9	0.88	0.76	0.85	0.83	0.91

Supplementary Figure 2. Accuracy metrics for AIMS models using histopathological assessment (AC/SqCC) as endpoint variable. Classification performance of the AIMS algorithm trained on seven individual training datasets plus a pooled training dataset (7DS) and applied to five test datasets: (**A**) Accuracy. (**B**) Balanced accuracy.

SFigure 3

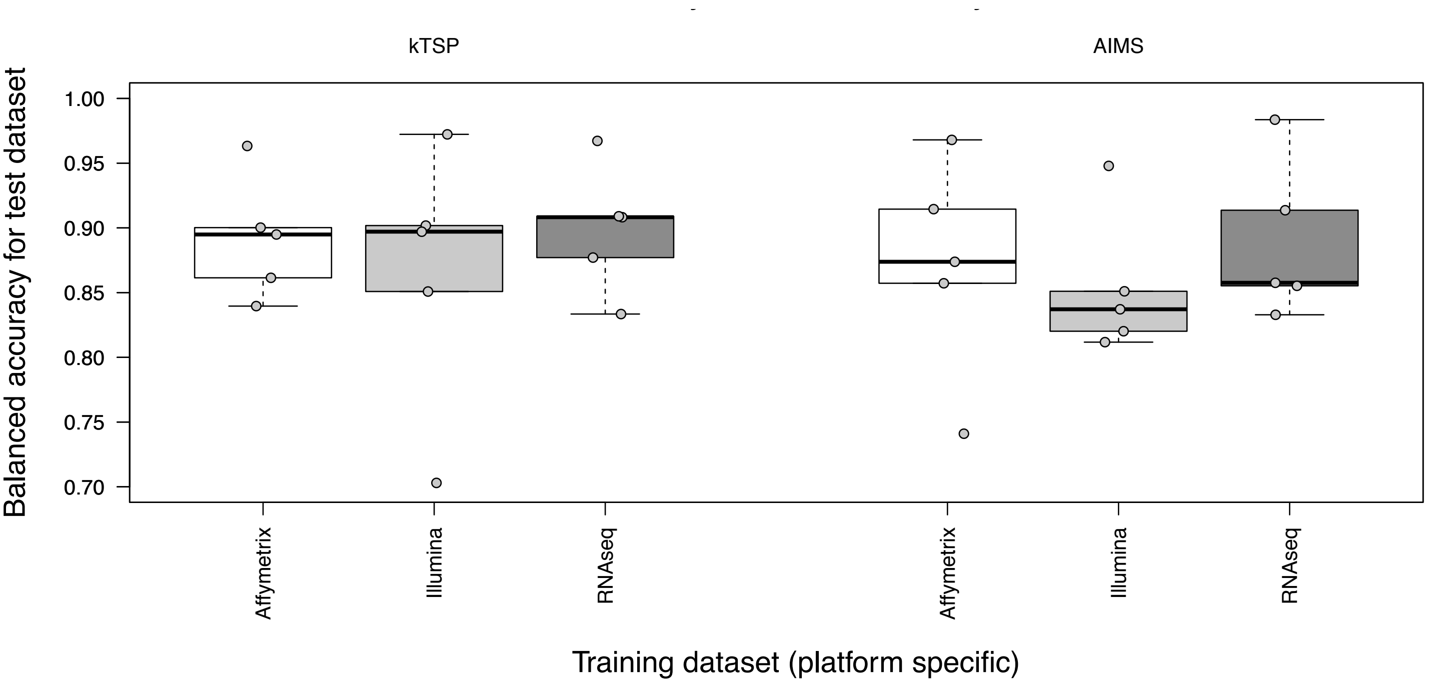

Supplementary Figure 3. Classification performance measured as balanced accuracy for kTSP and AIMS models in test datasets stratified by training datasets platform for the histology case study arm.

SFigure 4

A)

Accuracy for kTSP models

Training dataset

Test dataset	Sato et al.	Der et al.	Wilkerson et al.	TCGA	Shedden et al.	Fouret et al.	Okayama et al.	7DS
N=	183	127	116	230	444	103	226	1429
#TSP=	17	15	10	7	20	10	20	36
Botling et al.	0.91	0.87	0.91	0.73	0.89	0.91	0.77	0.92
Bhattacharjee et al.	0.81	0.37	0.43	0.62	0.43	0.38	0.71	0.44
Tarca et al.	0.83	0.79	0.82	0.64	0.78	0.83	0.7	0.81
Rousseaux et al.	0.88	0.89	0.85	0.82	0.87	0.88	0.88	0.91
Tomida et al.	0.85	0.83	0.75	0.78	0.9	0.66	0.9	0.75
Chitale et al.	0.84	0.81	0.67	0.85	0.77	0.78	0.88	0.8

B)

Balanced accuracy for kTSP models

Training dataset

Test dataset	Sato et al.	Der et al.	Wilkerson et al.	TCGA	Shedden et al.	Fouret et al.	Okayama et al.	7DS
N=	183	127	116	230	444	103	226	1429
#TSP=	17	15	10	7	20	10	20	36
Botling et al.	0.87	0.82	0.89	0.61	0.86	0.88	0.68	0.88
Bhattacharjee et al.	0.8	0.5	0.55	0.69	0.55	0.51	0.74	0.55
Tarca et al.	0.8	0.75	0.79	0.56	0.74	0.8	0.65	0.77
Rousseaux et al.	0.9	0.87	0.88	0.74	0.9	0.89	0.85	0.93
Tomida et al.	0.86	0.8	0.79	0.73	0.9	0.71	0.88	0.79
Chitale et al.	0.87	0.84	0.72	0.85	0.81	0.81	0.88	0.83

Supplementary Figure 4. Accuracy metrics for kTSP models using molecular subtypes (TRU/ non-TRU) as endpoint variable. Classification performance of the kTSP algorithm trained on seven individual training datasets plus a pooled training dataset (7DS) and applied to six test datasets: (**A**) Accuracy. (**B**) Balanced accuracy.

SFigure 5

A)

Accuracy for AIMS models

Training dataset

Test dataset	Sato et al.	Der et al.	Wilkerson et al.	TCGA	Shedden et al.	Fouret et al.	Okayama et al.	7DS
N=	183	127	116	230	444	103	226	1429
#TSP=	4	10	49	4	50	19	48	33
Botling et al.	0.8	0.76	0.91	0.74	0.89	0.89	0.75	0.89
Bhattacharjee et al.	0.69	0.41	0.38	0.44	0.68	0.38	0.67	0.44
Tarca et al.	0.78	0.7	0.82	0.65	0.78	0.78	0.69	0.78
Rousseaux et al.	0.76	0.84	0.87	0.84	0.89	0.92	0.88	0.88
Tomida et al.	0.7	0.74	0.86	0.62	0.91	0.85	0.85	0.91
Chitale et al.	0.83	0.83	0.71	0.83	0.85	0.81	0.81	0.9

B)

Balanced accuracy for AIMS models

Training dataset

Test dataset	Sato et al.	Der et al.	Wilkerson et al.	TCGA	Shedden et al.	Fouret et al.	Okayama et al.	7DS
N=	183	127	116	230	444	103	226	1429
#TSP=	4	10	49	4	50	19	48	33
Botling et al.	0.79	0.66	0.89	0.62	0.84	0.84	0.65	0.84
Bhattacharjee et al.	0.74	0.53	0.51	0.55	0.75	0.51	0.56	0.55
Tarca et al.	0.74	0.64	0.79	0.58	0.74	0.74	0.62	0.74
Rousseaux et al.	0.75	0.78	0.9	0.76	0.89	0.91	0.83	0.87
Tomida et al.	0.63	0.68	0.89	0.52	0.91	0.83	0.82	0.92
Chitale et al.	0.82	0.85	0.75	0.84	0.87	0.84	0.8	0.91

Supplementary Figure 5. Accuracy metrics for AIMS models using molecular subtypes (TRU/non-TRU) as endpoint variable. Classification performance of the AIMS algorithm trained on seven individual training datasets plus a pooled training dataset (7DS) and applied to six test datasets: (**A**) Accuracy. (**B**) Balanced accuracy.

SFigure 6

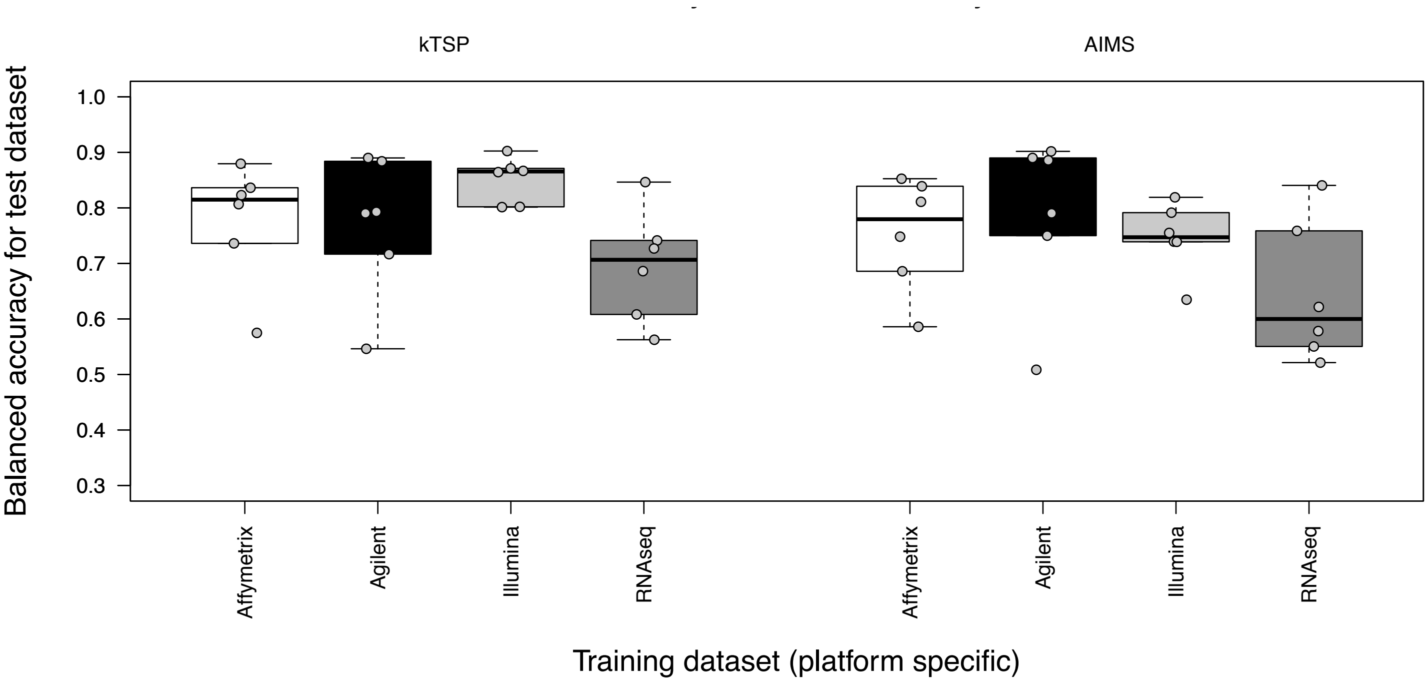

Supplementary Figure 6. Classification performance measured as balanced accuracy for kTSP and AIMS models in test datasets stratified by training datasets platform for the molecular subtype case study arm.

Supplementary Figure 7. Gene expression ratios for TSP/decision rules behind the kTSP model built from the pooled training dataset (7DS) for the histology case study arm. Results shown in 7DS and GSE30219 datasets. Red=SqCC, blue=AC.

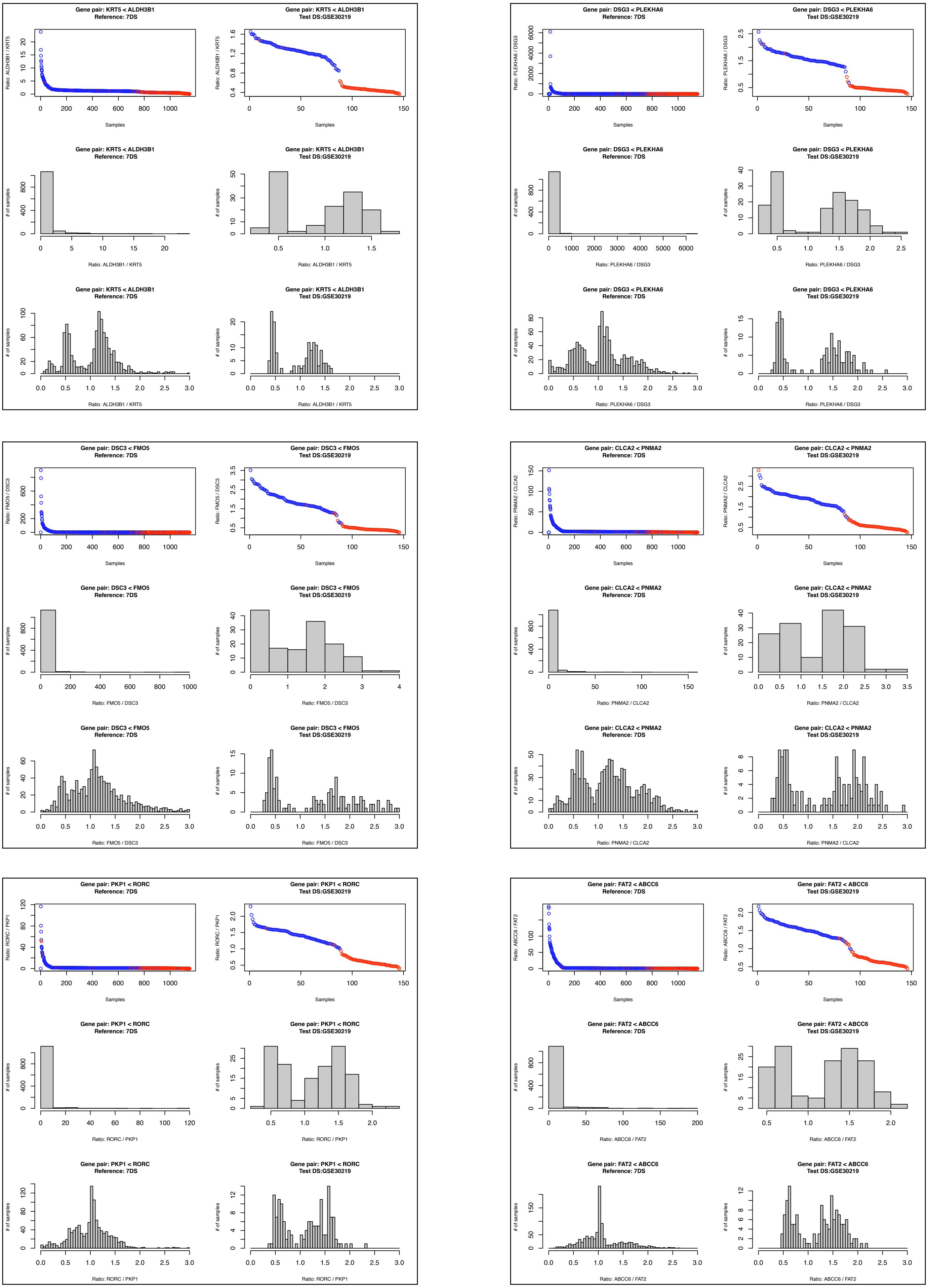

Supplementary Figure 7. Gene expression ratios for TSP/decision rules behind the kTSP model built from the pooled training dataset (7DS) for the histology case study arm. Results shown in 7DS and GSE30219 datasets - CONTINUED

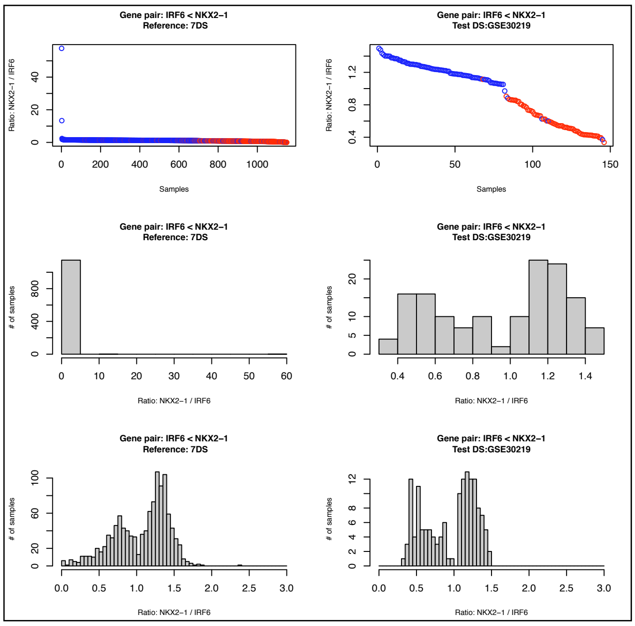

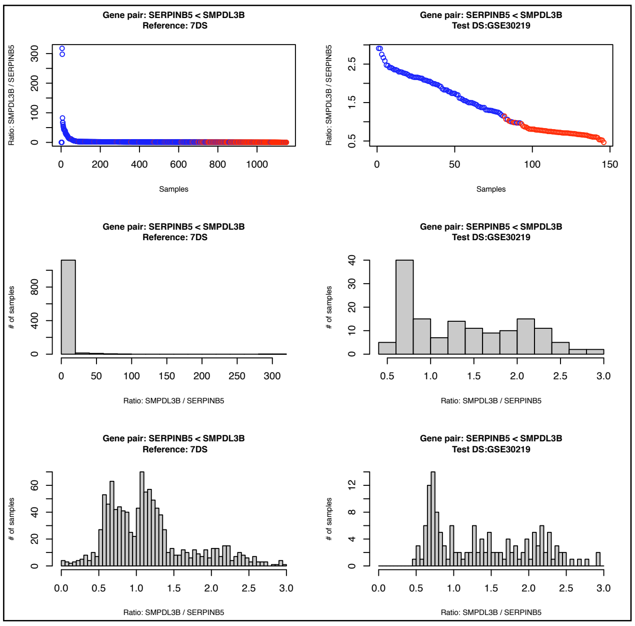

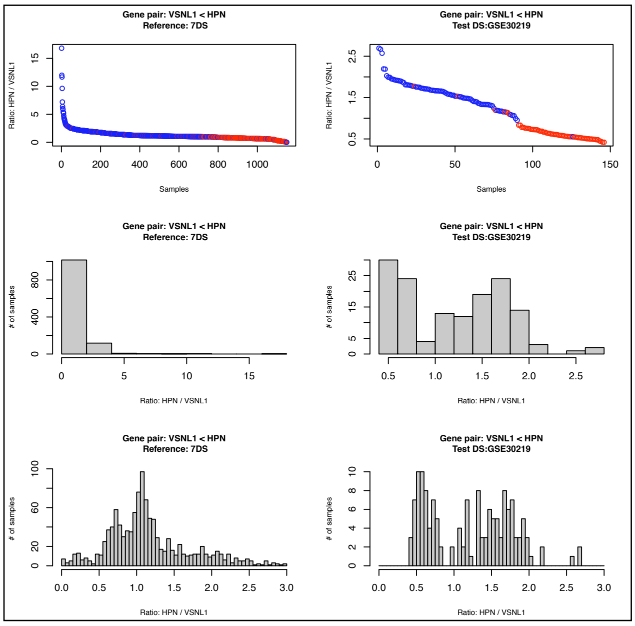

Supplementary Figure 8. Gene expression ratios for TSP/decision rules behind the AIMS model built from the pooled training dataset (7DS) for the histology case study arm. Results shown in 7DS and GSE30219 datasets. Red=SqCC, blue=AC.

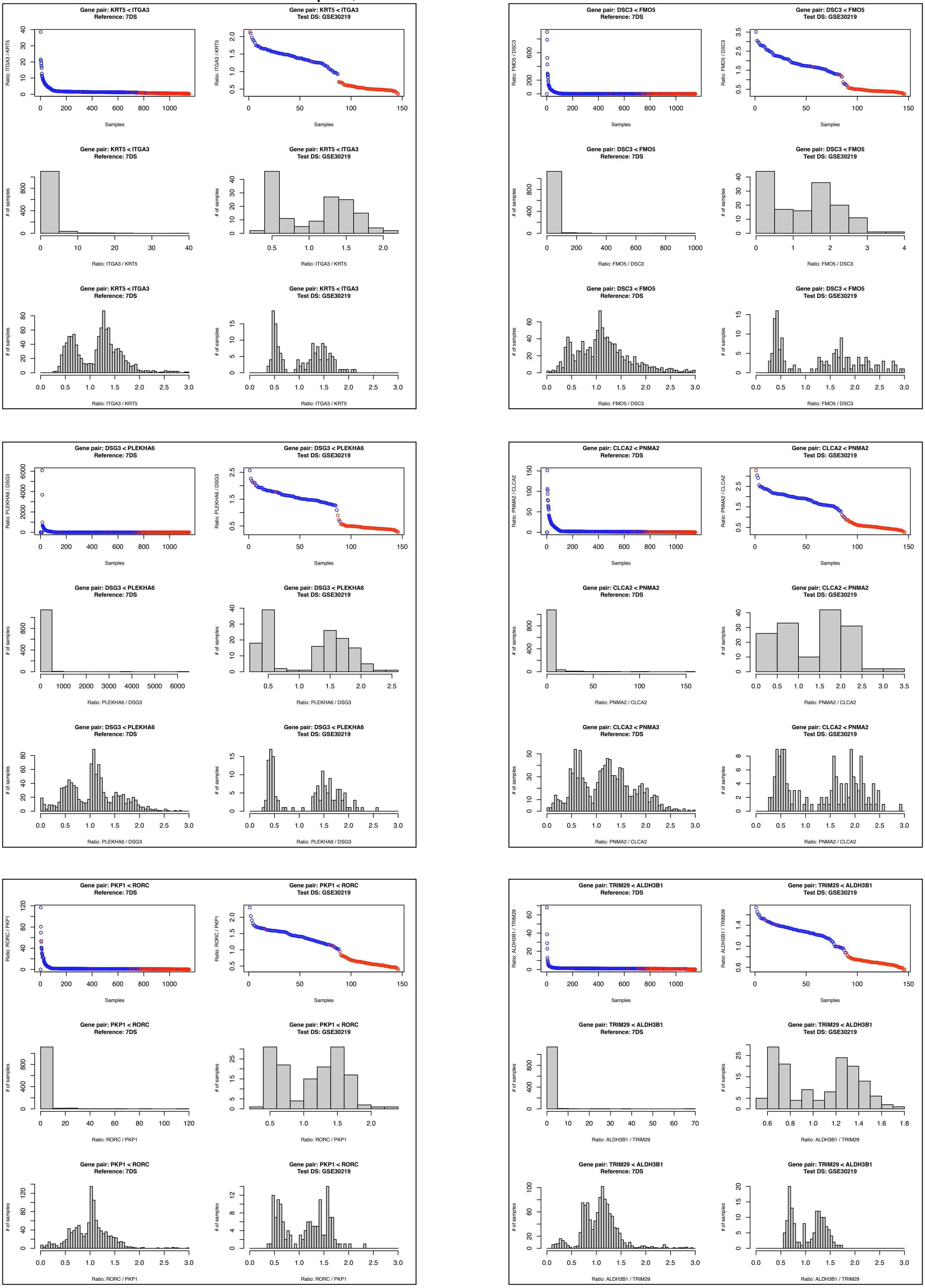

Supplementary Figure 9. Gene expression ratios for TSP/decision rules behind the kTSP model built from the pooled training dataset (7DS) for the molecular subtype case study arm. Results shown in 7DS and GSE30219 datasets. Red=non-TRU, blue=TRU.

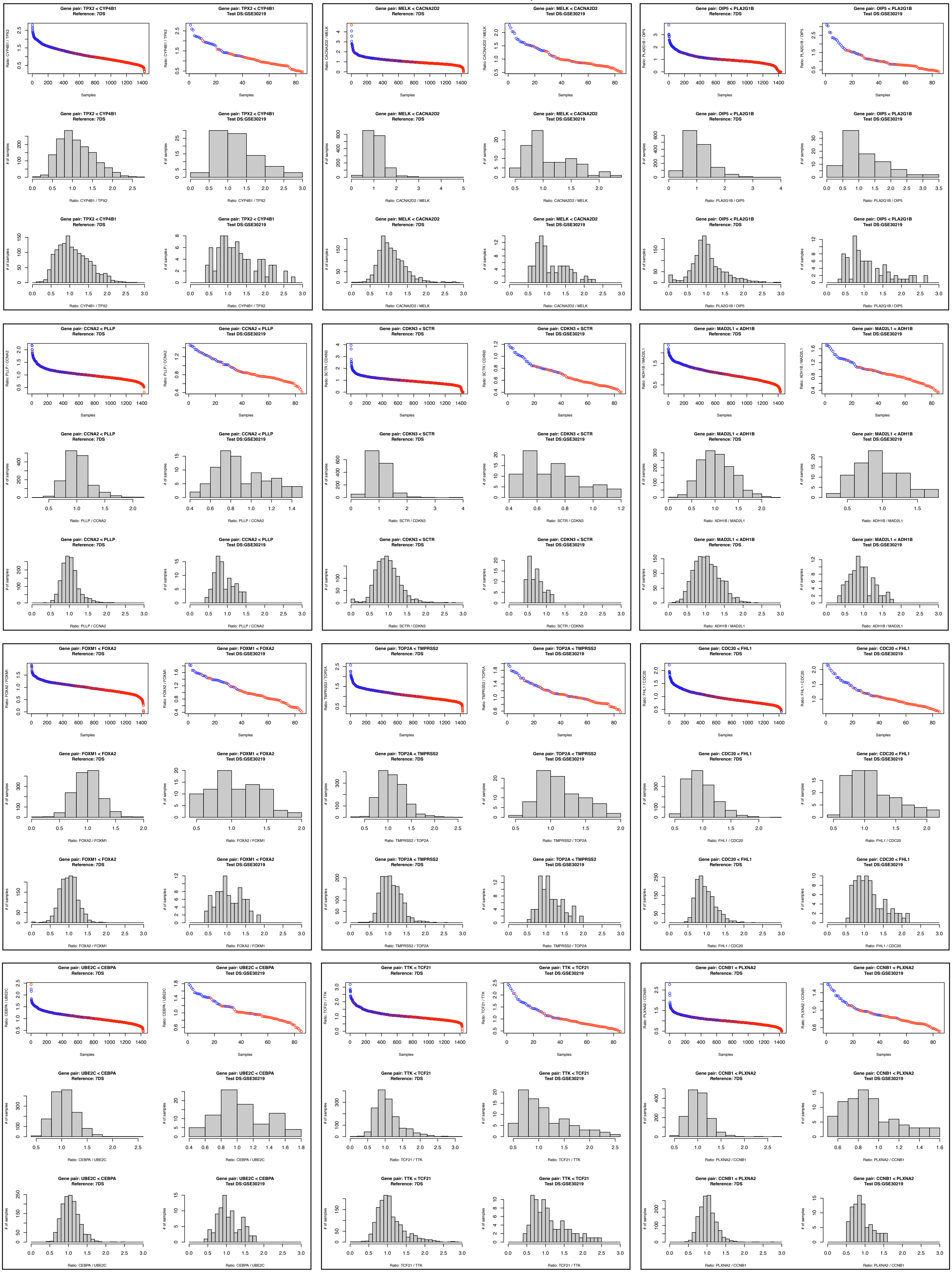

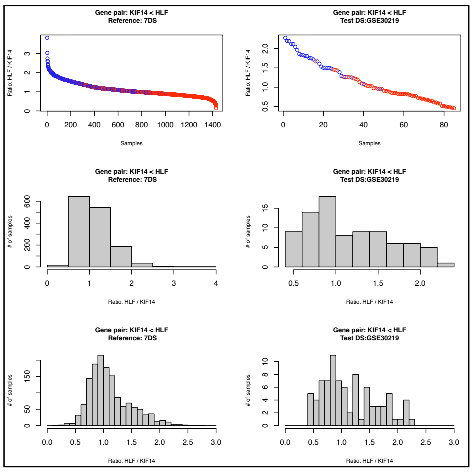
Gene pair: KIF14 < HLF
Reference: 7DS
Test DS:GSE30219
Ratio: HLF / KIF14
Samples
# of samples

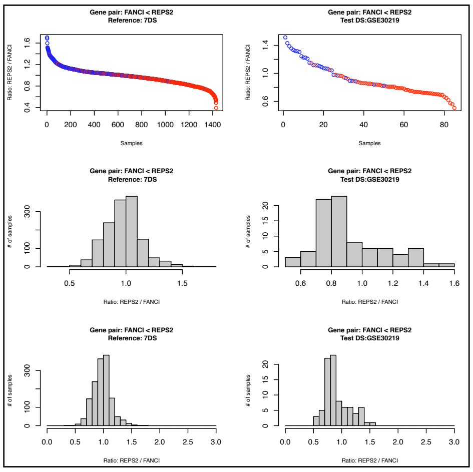
Gene pair: FANCI < REPS2
Reference: 7DS
Test DS:GSE30219
Ratio: REPS2 / FANCI
Samples
# of samples

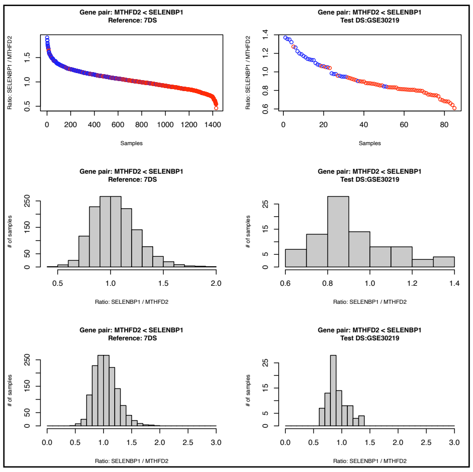
Gene pair: MTHFD2 < SELENBP1
Reference: 7DS
Test DS:GSE30219
Ratio: SELENBP1 / MTHFD2
Samples
# of samples

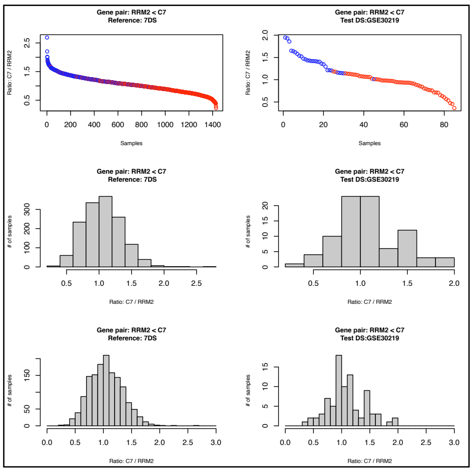
Gene pair: RRM2 < C7
Reference: 7DS
Test DS:GSE30219
Ratio: C7 / RRM2
Samples
# of samples

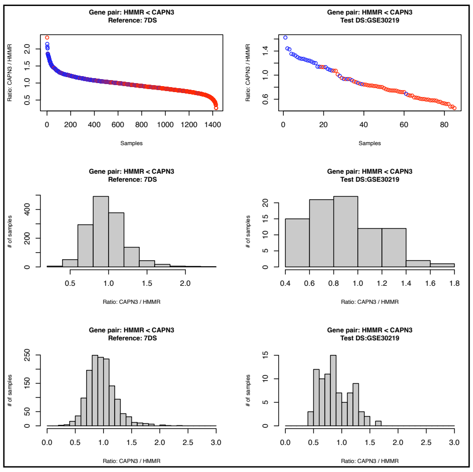
Gene pair: HMMR < CAPN3
Reference: 7DS
Test DS:GSE30219
Ratio: CAPN3 / HMMR
Samples
# of samples

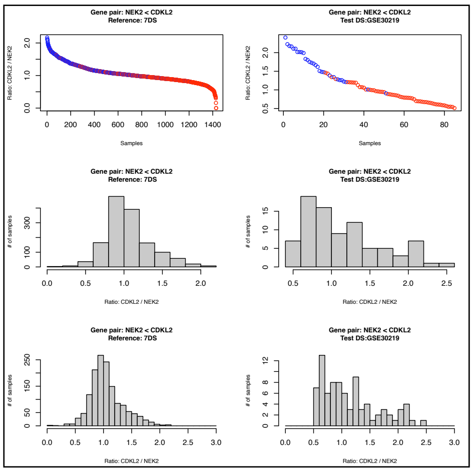
Gene pair: NEK2 < CDKL2
Reference: 7DS
Test DS:GSE30219
Ratio: CDKL2 / NEK2
Samples
# of samples

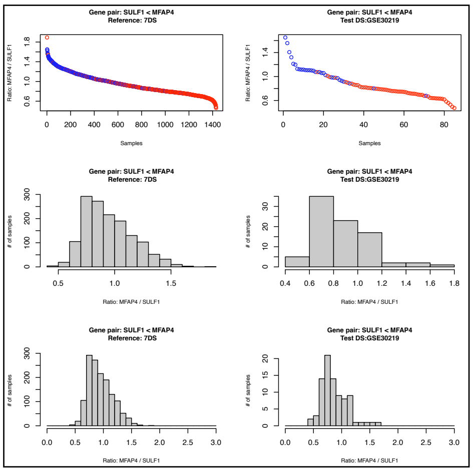
Gene pair: SULF1 < MFAP4
Reference: 7DS
Test DS:GSE30219
Ratio: MFAP4 / SULF1
Samples
# of samples

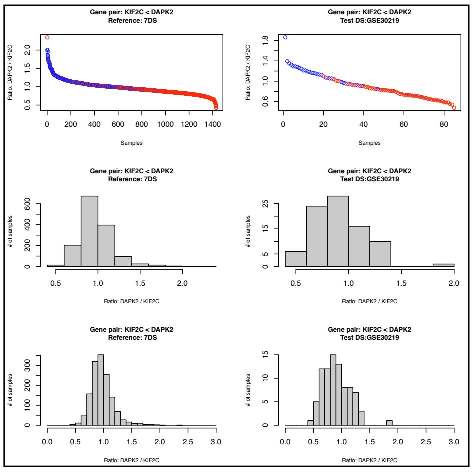
Gene pair: KIF2C < DAPK2
Reference: 7DS
Test DS:GSE30219
Ratio: DAPK2 / KIF2C
Samples
# of samples

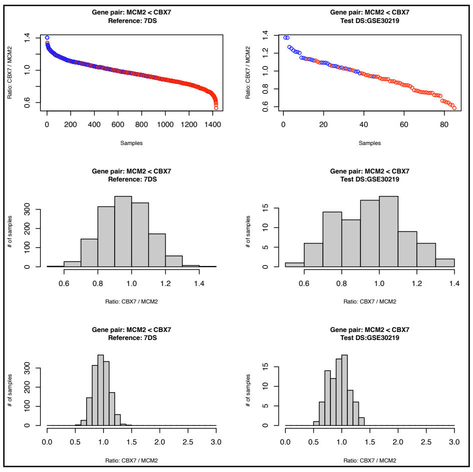
Gene pair: MCM2 < CBX7
Reference: 7DS
Test DS:GSE30219
Ratio: CBX7 / MCM2
Samples
# of samples

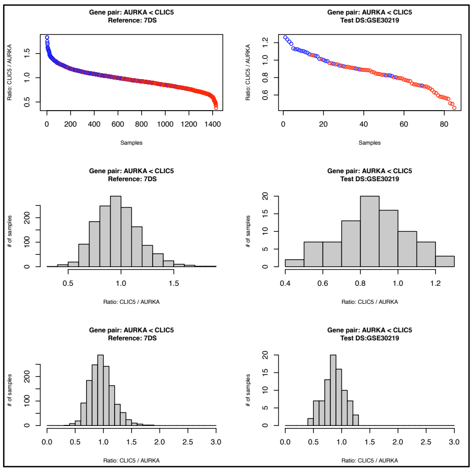
Gene pair: AURKA < CLIC5
Reference: 7DS
Test DS:GSE30219
Ratio: CLIC5 / AURKA
Samples
# of samples

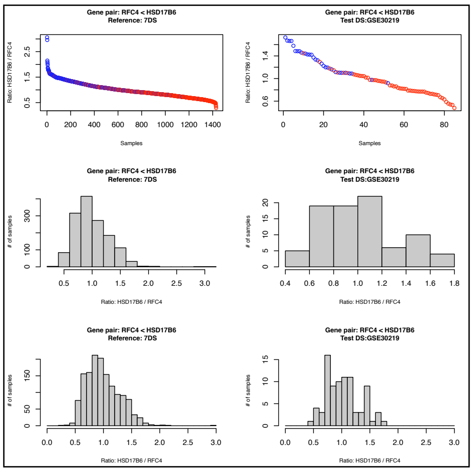
Gene pair: RFC4 < HSD17B6
Reference: 7DS
Test DS:GSE30219
Ratio: HSD17B6 / RFC4
Samples
# of samples

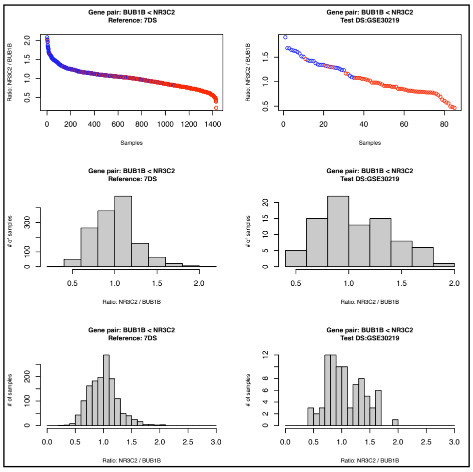
Gene pair: BUB1B < NR3C2
Reference: 7DS
Test DS:GSE30219
Ratio: NR3C2 / BUB1B
Samples
# of samples

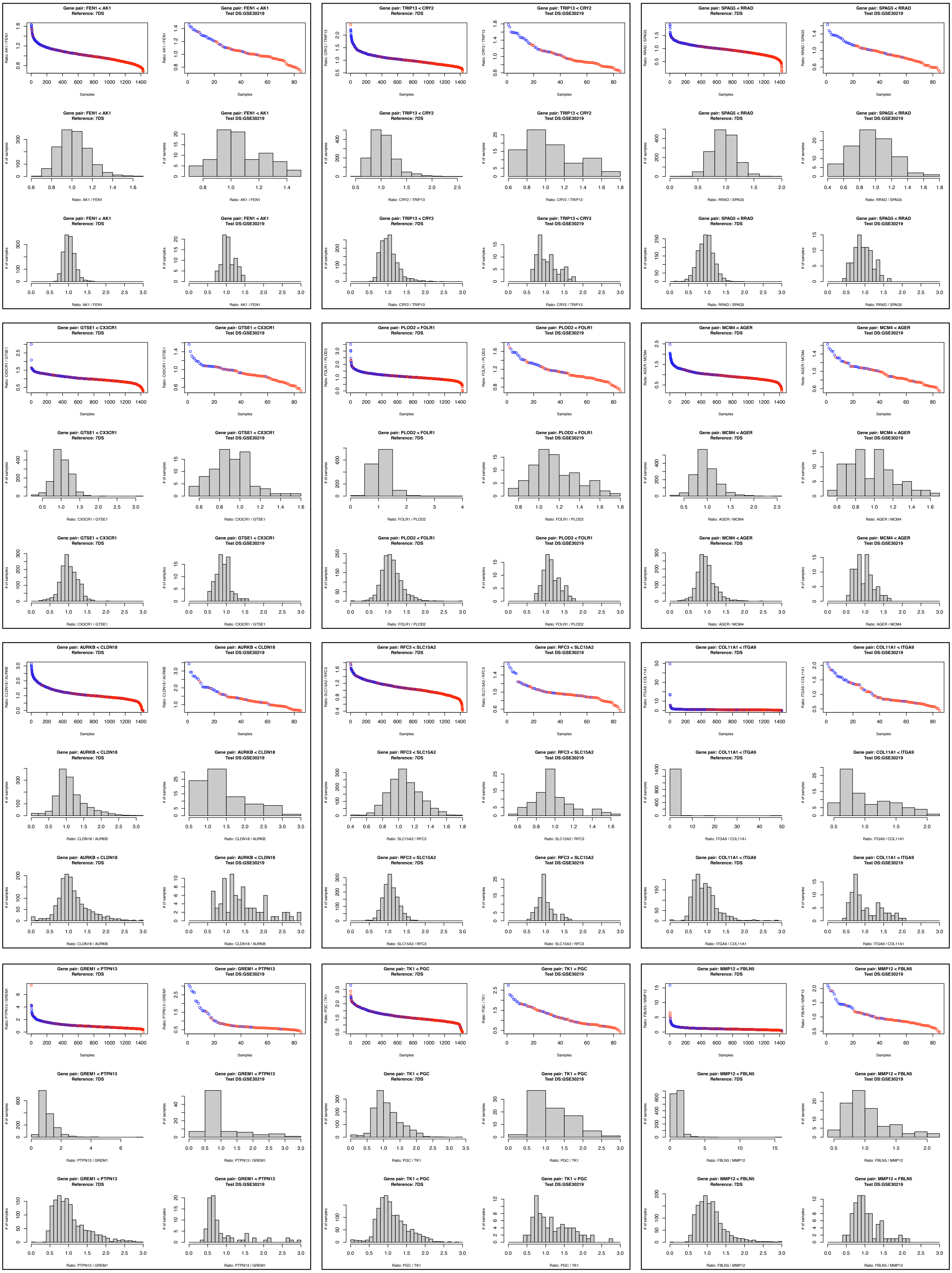

Gene pair: FEN1 < AK1
Reference: 7DS
Test DS:GSE30219
Gene pair: TRIP13 < CRY2
Reference: 7DS
Test DS:GSE30219
Gene pair: SPAG5 < RRAD
Reference: 7DS
Test DS:GSE30219
Gene pair: GTSE1 < CX3CR1
Reference: 7DS
Test DS:GSE30219
Gene pair: PLOD2 < FOLR1
Reference: 7DS
Test DS:GSE30219
Gene pair: MCM4 < AGER
Reference: 7DS
Test DS:GSE30219
Gene pair: AURKB < CLDN18
Reference: 7DS
Test DS:GSE30219
Gene pair: RFC3 < SLC15A2
Reference: 7DS
Test DS:GSE30219
Gene pair: COL11A1 < ITGA9
Reference: 7DS
Test DS:GSE30219
Gene pair: GREM1 < PTPN13
Reference: 7DS
Test DS:GSE30219
Gene pair: TK1 < PGC
Reference: 7DS
Test DS:GSE30219
Gene pair: MMP12 < FBLN5
Reference: 7DS
Test DS:GSE30219

Supplementary Figure 10. Gene expression ratios for TSP/decision rules behind the AIMS model built from the pooled training dataset (7DS) for the molecular subtype case study arm. Results shown in 7DS and GSE30219 datasets. Red=non-TRU, blue=TRU.

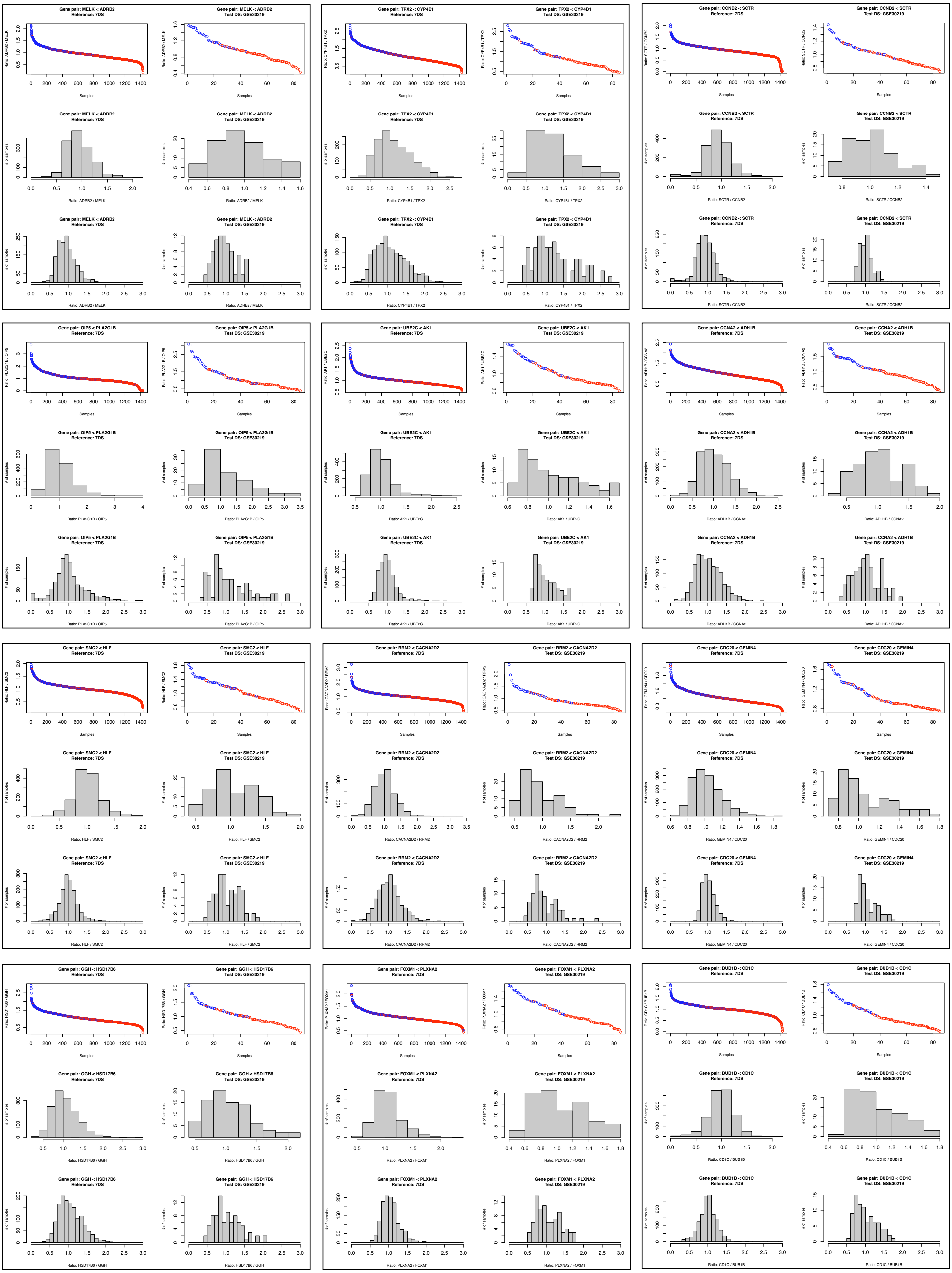

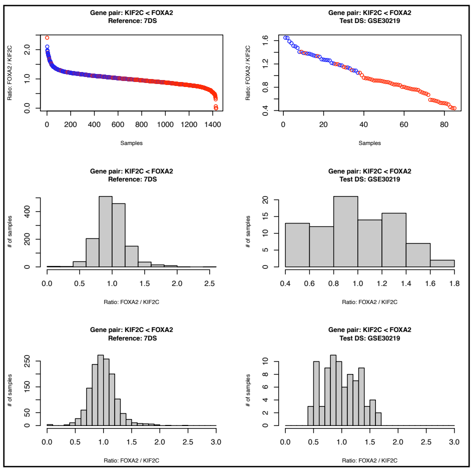
Gene pair: KIF2C < FOXA2
Reference: 7DS
Ratio: FOXA2 / KIF2C
Samples
Gene pair: KIF2C < FOXA2
Test DS: GSE30219
Ratio: FOXA2 / KIF2C
Samples
Gene pair: KIF2C < FOXA2
Reference: 7DS
# of samples
Ratio: FOXA2 / KIF2C
Gene pair: KIF2C < FOXA2
Test DS: GSE30219
# of samples
Ratio: FOXA2 / KIF2C
Gene pair: KIF2C < FOXA2
Reference: 7DS
# of samples
Ratio: FOXA2 / KIF2C
Gene pair: KIF2C < FOXA2
Test DS: GSE30219
# of samples
Ratio: FOXA2 / KIF2C

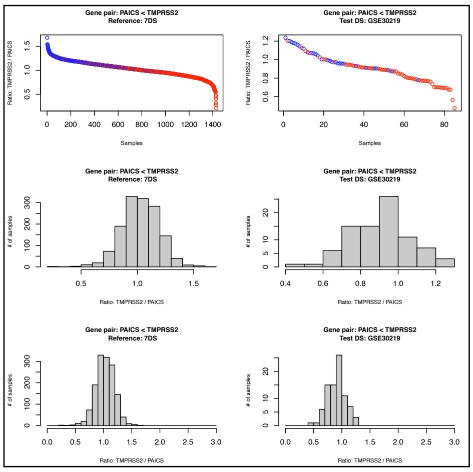
Gene pair: PAICS < TMPRSS2
Reference: 7DS
Ratio: TMPRSS2 / PAICS
Samples
Gene pair: PAICS < TMPRSS2
Test DS: GSE30219
Ratio: TMPRSS2 / PAICS
Samples
Gene pair: PAICS < TMPRSS2
Reference: 7DS
# of samples
Ratio: TMPRSS2 / PAICS
Gene pair: PAICS < TMPRSS2
Test DS: GSE30219
# of samples
Ratio: TMPRSS2 / PAICS
Gene pair: PAICS < TMPRSS2
Reference: 7DS
# of samples
Ratio: TMPRSS2 / PAICS
Gene pair: PAICS < TMPRSS2
Test DS: GSE30219
# of samples
Ratio: TMPRSS2 / PAICS

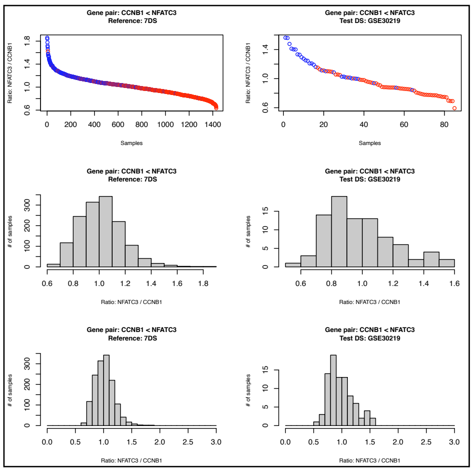
Gene pair: CCNB1 < NFATC3
Reference: 7DS
Ratio: NFATC3 / CCNB1
Samples
Gene pair: CCNB1 < NFATC3
Test DS: GSE30219
Ratio: NFATC3 / CCNB1
Samples
Gene pair: CCNB1 < NFATC3
Reference: 7DS
# of samples
Ratio: NFATC3 / CCNB1
Gene pair: CCNB1 < NFATC3
Test DS: GSE30219
# of samples
Ratio: NFATC3 / CCNB1
Gene pair: CCNB1 < NFATC3
Reference: 7DS
# of samples
Ratio: NFATC3 / CCNB1
Gene pair: CCNB1 < NFATC3
Test DS: GSE30219
# of samples
Ratio: NFATC3 / CCNB1

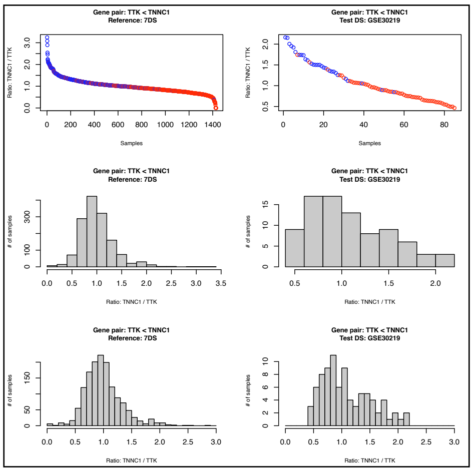
Gene pair: TTK < TNNC1
Reference: 7DS
Ratio: TNNC1 / TTK
Samples
Gene pair: TTK < TNNC1
Test DS: GSE30219
Ratio: TNNC1 / TTK
Samples
Gene pair: TTK < TNNC1
Reference: 7DS
# of samples
Ratio: TNNC1 / TTK
Gene pair: TTK < TNNC1
Test DS: GSE30219
# of samples
Ratio: TNNC1 / TTK
Gene pair: TTK < TNNC1
Reference: 7DS
# of samples
Ratio: TNNC1 / TTK
Gene pair: TTK < TNNC1
Test DS: GSE30219
# of samples
Ratio: TNNC1 / TTK

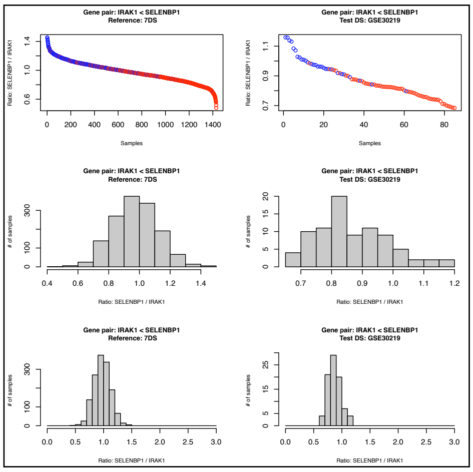
Gene pair: IRAK1 < SELENBP1
Reference: 7DS
Ratio: SELENBP1 / IRAK1
Samples
Gene pair: IRAK1 < SELENBP1
Test DS: GSE30219
Ratio: SELENBP1 / IRAK1
Samples
Gene pair: IRAK1 < SELENBP1
Reference: 7DS
# of samples
Ratio: SELENBP1 / IRAK1
Gene pair: IRAK1 < SELENBP1
Test DS: GSE30219
# of samples
Ratio: SELENBP1 / IRAK1
Gene pair: IRAK1 < SELENBP1
Reference: 7DS
# of samples
Ratio: SELENBP1 / IRAK1
Gene pair: IRAK1 < SELENBP1
Test DS: GSE30219
# of samples
Ratio: SELENBP1 / IRAK1

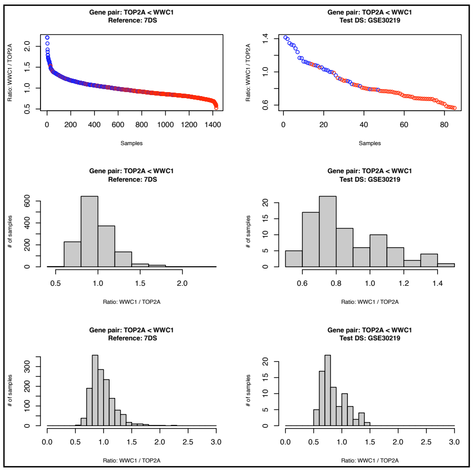
Gene pair: TOP2A < WWC1
Reference: 7DS
Ratio: WWC1 / TOP2A
Samples
Gene pair: TOP2A < WWC1
Test DS: GSE30219
Ratio: WWC1 / TOP2A
Samples
Gene pair: TOP2A < WWC1
Reference: 7DS
# of samples
Ratio: WWC1 / TOP2A
Gene pair: TOP2A < WWC1
Test DS: GSE30219
# of samples
Ratio: WWC1 / TOP2A
Gene pair: TOP2A < WWC1
Reference: 7DS
# of samples
Ratio: WWC1 / TOP2A
Gene pair: TOP2A < WWC1
Test DS: GSE30219
# of samples
Ratio: WWC1 / TOP2A

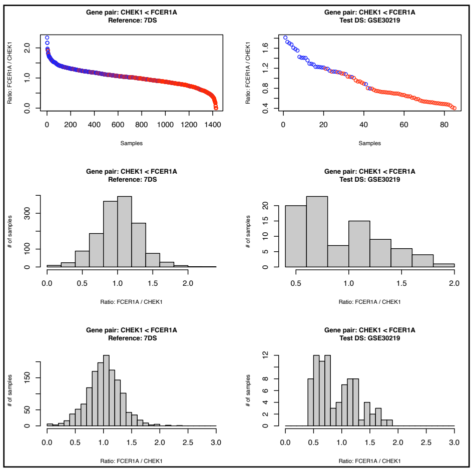
Gene pair: CHEK1 < FCER1A
Reference: 7DS
Ratio: FCER1A / CHEK1
Samples
Gene pair: CHEK1 < FCER1A
Test DS: GSE30219
Ratio: FCER1A / CHEK1
Samples
Gene pair: CHEK1 < FCER1A
Reference: 7DS
# of samples
Ratio: FCER1A / CHEK1
Gene pair: CHEK1 < FCER1A
Test DS: GSE30219
# of samples
Ratio: FCER1A / CHEK1
Gene pair: CHEK1 < FCER1A
Reference: 7DS
# of samples
Ratio: FCER1A / CHEK1
Gene pair: CHEK1 < FCER1A
Test DS: GSE30219
# of samples
Ratio: FCER1A / CHEK1

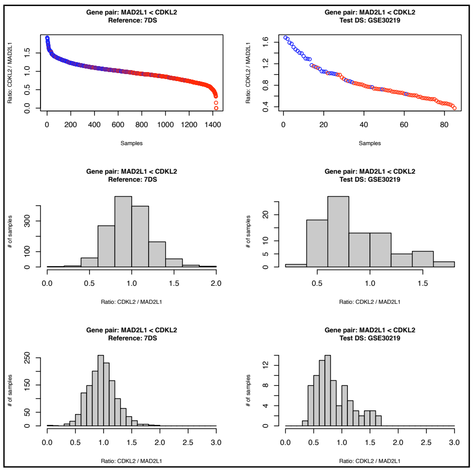
Gene pair: MAD2L1 < CDKL2
Reference: 7DS
Ratio: CDKL2 / MAD2L1
Samples
Gene pair: MAD2L1 < CDKL2
Test DS: GSE30219
Ratio: CDKL2 / MAD2L1
Samples
Gene pair: MAD2L1 < CDKL2
Reference: 7DS
# of samples
Ratio: CDKL2 / MAD2L1
Gene pair: MAD2L1 < CDKL2
Test DS: GSE30219
# of samples
Ratio: CDKL2 / MAD2L1
Gene pair: MAD2L1 < CDKL2
Reference: 7DS
# of samples
Ratio: CDKL2 / MAD2L1
Gene pair: MAD2L1 < CDKL2
Test DS: GSE30219
# of samples
Ratio: CDKL2 / MAD2L1

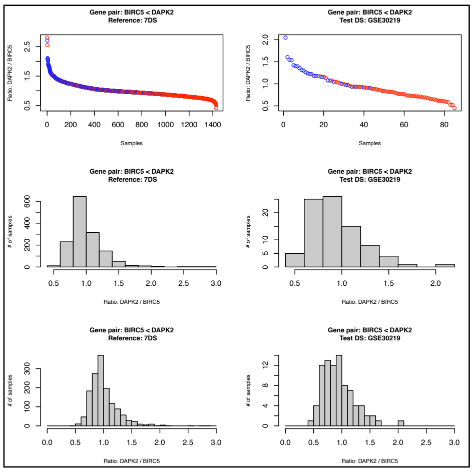
Gene pair: BIRC5 < DAPK2
Reference: 7DS
Ratio: DAPK2 / BIRC5
Samples
Gene pair: BIRC5 < DAPK2
Test DS: GSE30219
Ratio: DAPK2 / BIRC5
Samples
Gene pair: BIRC5 < DAPK2
Reference: 7DS
# of samples
Ratio: DAPK2 / BIRC5
Gene pair: BIRC5 < DAPK2
Test DS: GSE30219
# of samples
Ratio: DAPK2 / BIRC5
Gene pair: BIRC5 < DAPK2
Reference: 7DS
# of samples
Ratio: DAPK2 / BIRC5
Gene pair: BIRC5 < DAPK2
Test DS: GSE30219
# of samples
Ratio: DAPK2 / BIRC5

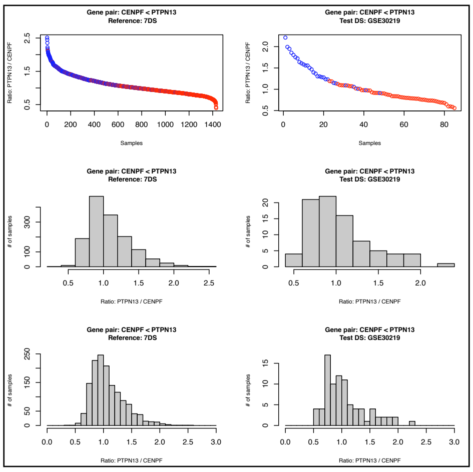
Gene pair: CENPF < PTPN13
Reference: 7DS
Ratio: PTPN13 / CENPF
Samples
Gene pair: CENPF < PTPN13
Test DS: GSE30219
Ratio: PTPN13 / CENPF
Samples
Gene pair: CENPF < PTPN13
Reference: 7DS
# of samples
Ratio: PTPN13 / CENPF
Gene pair: CENPF < PTPN13
Test DS: GSE30219
# of samples
Ratio: PTPN13 / CENPF
Gene pair: CENPF < PTPN13
Reference: 7DS
# of samples
Ratio: PTPN13 / CENPF
Gene pair: CENPF < PTPN13
Test DS: GSE30219
# of samples
Ratio: PTPN13 / CENPF

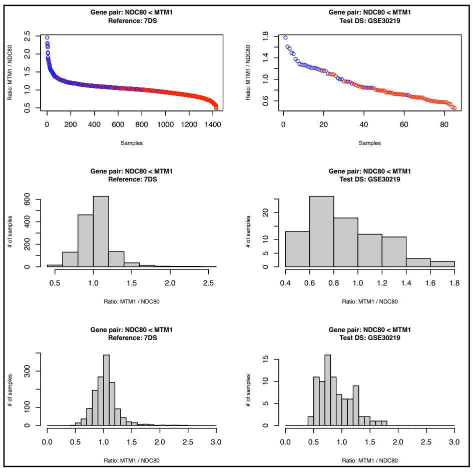
Gene pair: NDC80 < MTM1
Reference: 7DS
Ratio: MTM1 / NDC80
Samples
Gene pair: NDC80 < MTM1
Test DS: GSE30219
Ratio: MTM1 / NDC80
Samples
Gene pair: NDC80 < MTM1
Reference: 7DS
# of samples
Ratio: MTM1 / NDC80
Gene pair: NDC80 < MTM1
Test DS: GSE30219
# of samples
Ratio: MTM1 / NDC80
Gene pair: NDC80 < MTM1
Reference: 7DS
# of samples
Ratio: MTM1 / NDC80
Gene pair: NDC80 < MTM1
Test DS: GSE30219
# of samples
Ratio: MTM1 / NDC80

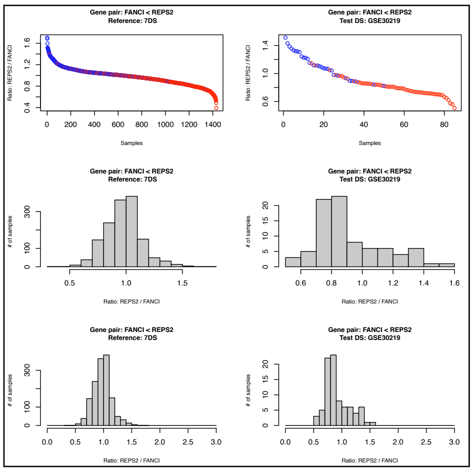
Gene pair: FANCI < REPS2
Reference: 7DS
Ratio: REPS2 / FANCI
Samples
Gene pair: FANCI < REPS2
Test DS: GSE30219
Ratio: REPS2 / FANCI
Samples
Gene pair: FANCI < REPS2
Reference: 7DS
# of samples
Ratio: REPS2 / FANCI
Gene pair: FANCI < REPS2
Test DS: GSE30219
# of samples
Ratio: REPS2 / FANCI
Gene pair: FANCI < REPS2
Reference: 7DS
# of samples
Ratio: REPS2 / FANCI
Gene pair: FANCI < REPS2
Test DS: GSE30219
# of samples
Ratio: REPS2 / FANCI

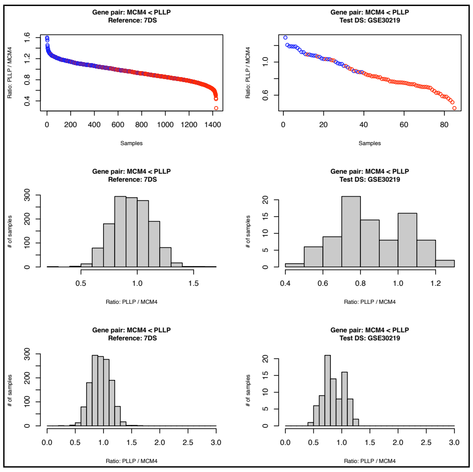
Gene pair: MCM4 < PLLP
Reference: 7DS
Test DS: GSE30219
Ratio: PLLP / MCM4
Samples
# of samples

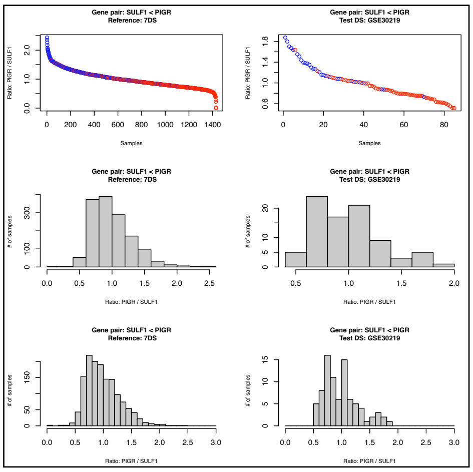
Gene pair: SULF1 < PIGR
Reference: 7DS
Test DS: GSE30219
Ratio: PIGR / SULF1
Samples
# of samples

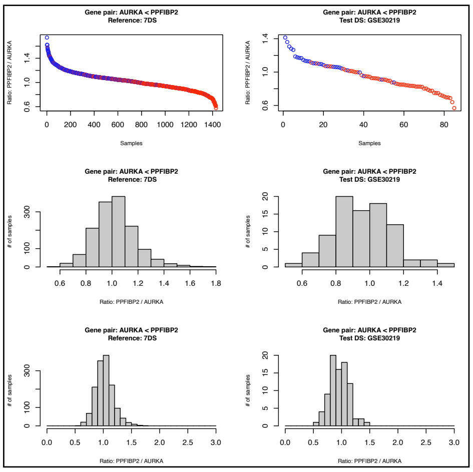
Gene pair: AURKA < PPFIBP2
Reference: 7DS
Test DS: GSE30219
Ratio: PPFIBP2 / AURKA
Samples
# of samples

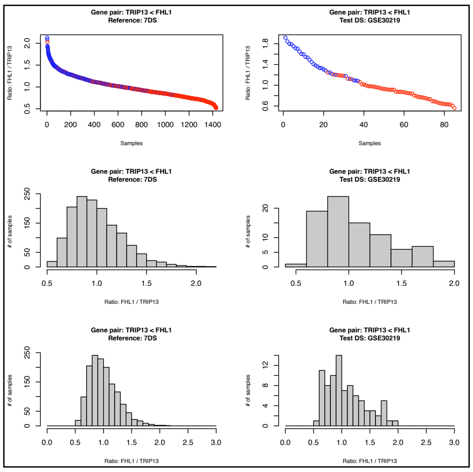
Gene pair: TRIP13 < FHL1
Reference: 7DS
Test DS: GSE30219
Ratio: FHL1 / TRIP13
Samples
# of samples

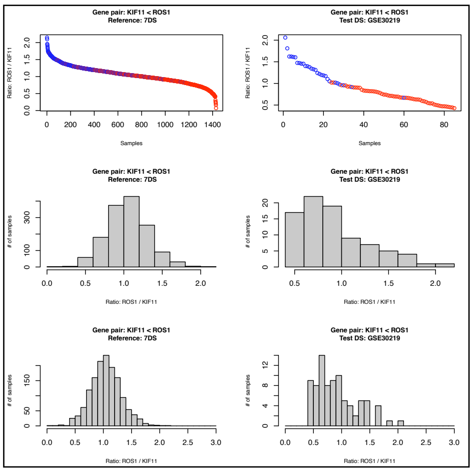
Gene pair: KIF11 < ROS1
Reference: 7DS
Test DS: GSE30219
Ratio: ROS1 / KIF11
Samples
# of samples

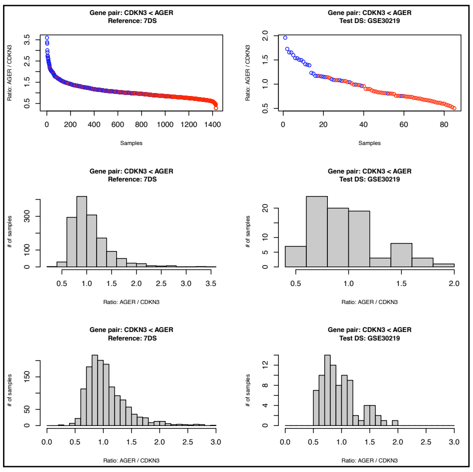
Gene pair: CDKN3 < AGER
Reference: 7DS
Test DS: GSE30219
Ratio: AGER / CDKN3
Samples
# of samples

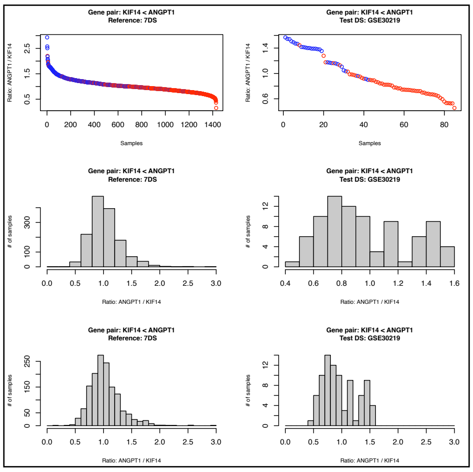
Gene pair: KIF14 < ANGPT1
Reference: 7DS
Test DS: GSE30219
Ratio: ANGPT1 / KIF14
Samples
# of samples

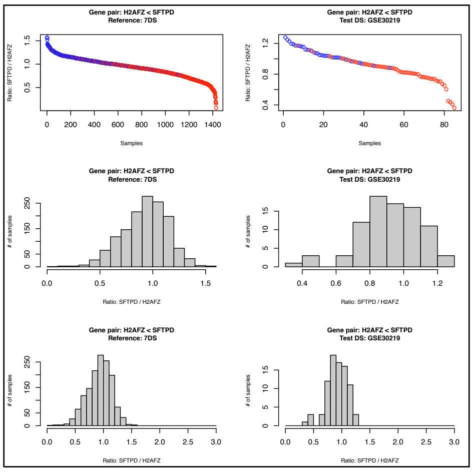
Gene pair: H2AFZ < SFTPD
Reference: 7DS
Test DS: GSE30219
Ratio: SFTPD / H2AFZ
Samples
# of samples

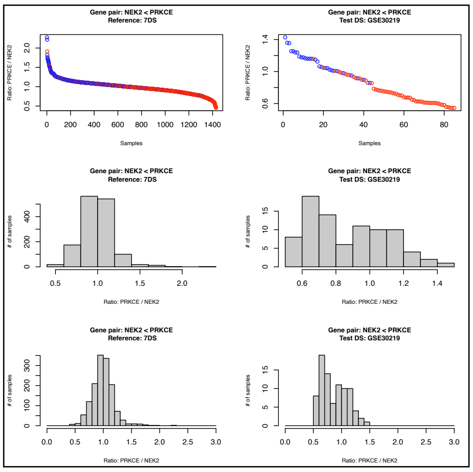
Gene pair: NEK2 < PRKCE
Reference: 7DS
Test DS: GSE30219
Ratio: PRKCE / NEK2
Samples
# of samples

SFigure 11.

A)

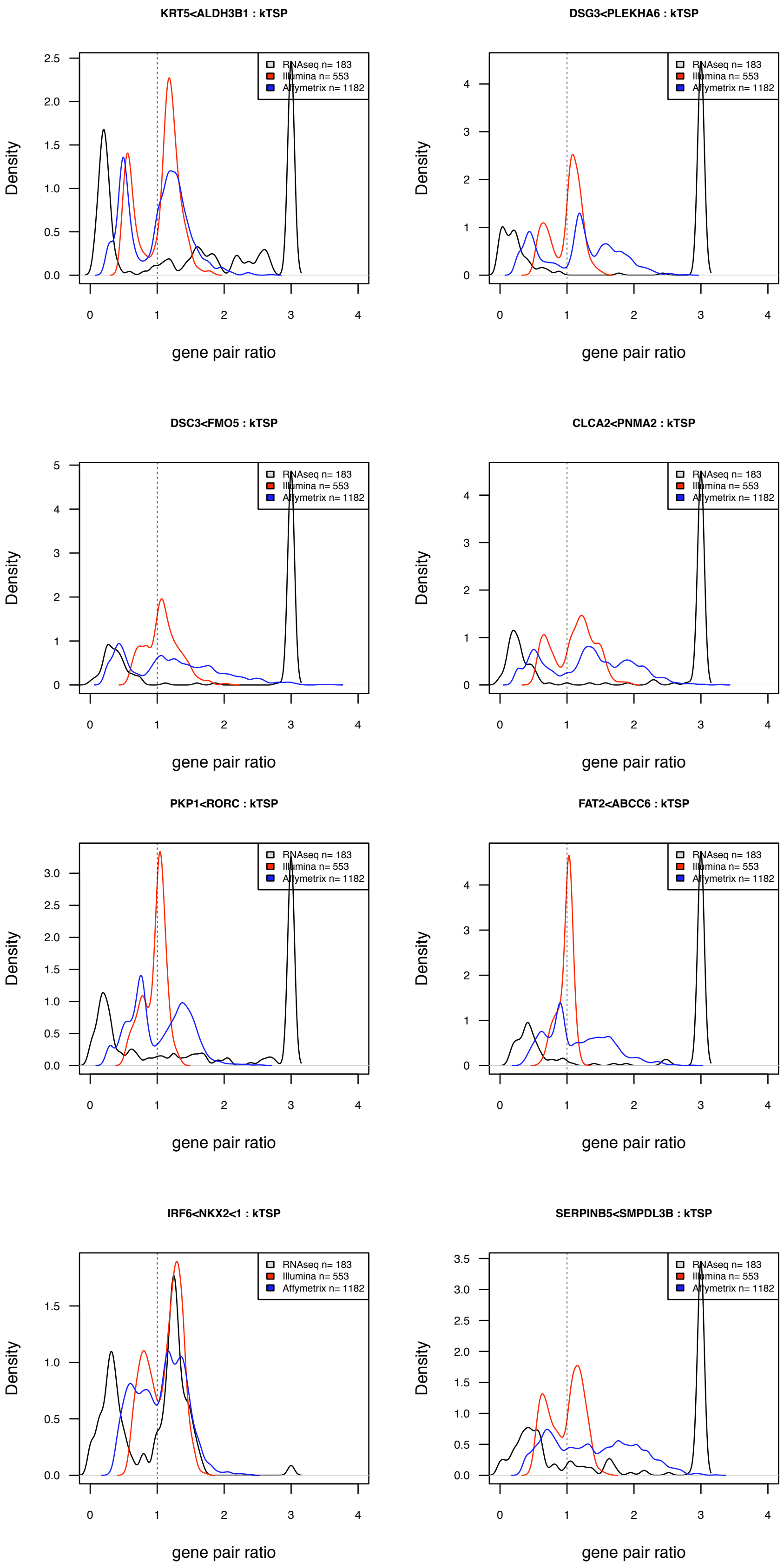

Supplementary Figure S11. Gene pair ratios versus gene expression platform. In all panels the SSPs were derived from the respective pooled 7DS training set and were applied to corresponding test datasets. Histograms per technical platform of the gene pair ratios from respective model were created by combining both the training and test samples. Histograms were tranferred to density plots using a kernel estimate function (density() in R). Panel legends show respective sample sizes. (**A**) Eight of nine gene pairs for the 7DS kTSP model in the histology case study arm stratified by technical platform. (**B**) All gene pairs for the 7DS AIMS model in the histological case study arm stratified by technical platform. (**C**) Eight of 36 gene pairs for the 7DS kTSP model in the molecular subtype case study arm stratified by technical platform. (**D**) Eight of 33 gene pairs for the 7DS AIMS model in the molecular subtype case study arm stratified by technical platform.

B)

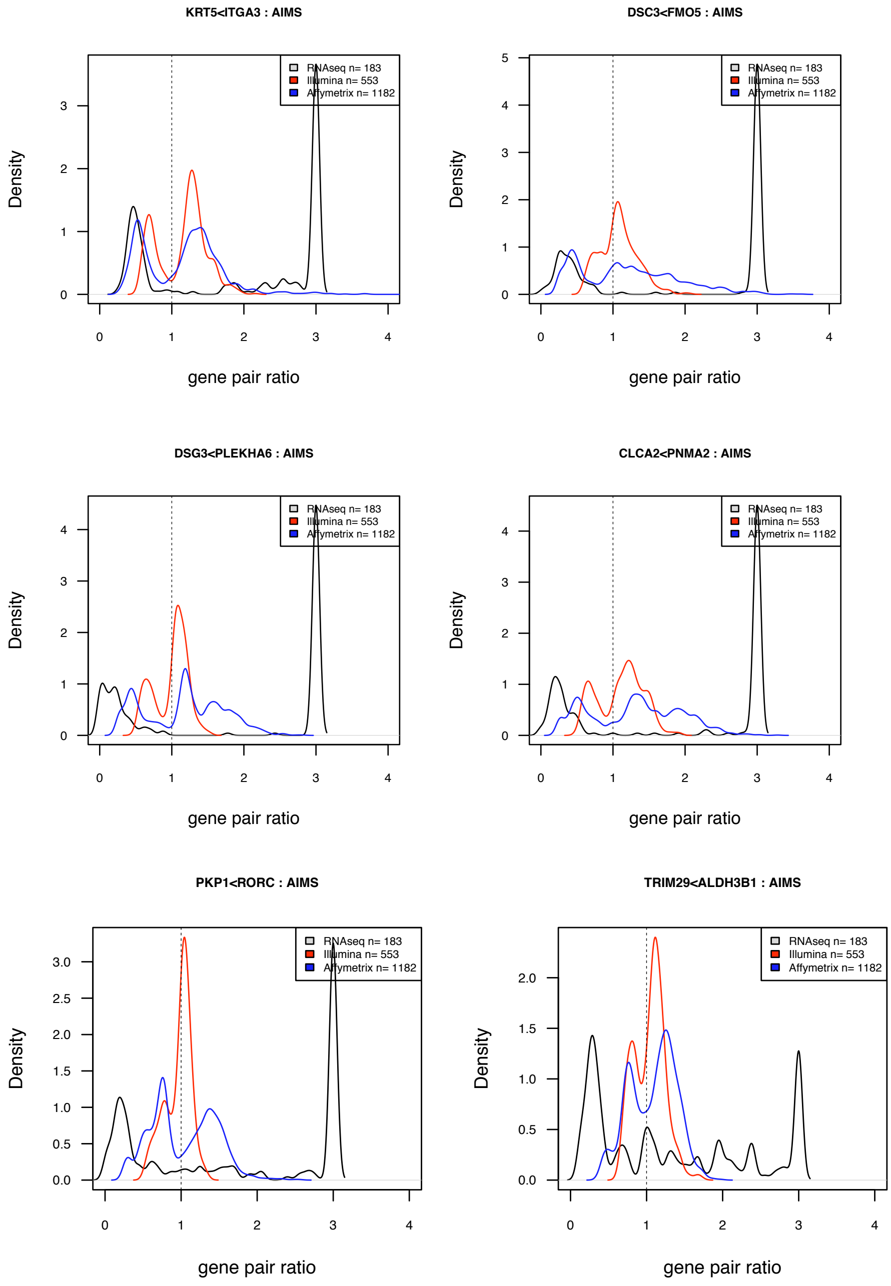
KRT5<ITGA3 : AIMS
DSC3<FMO5 : AIMS
DSG3<PLEKHA6 : AIMS
CLCA2<PNMA2 : AIMS
PKP1<RORC : AIMS
TRIM29<ALDH3B1 : AIMS
RNAseq n= 183
Illumina n= 553
Affymetrix n= 1182
Density
gene pair ratio
0
1
2
3
4
5
0.0
0.5
1.0
1.5
2.0
2.5
3.0

C)

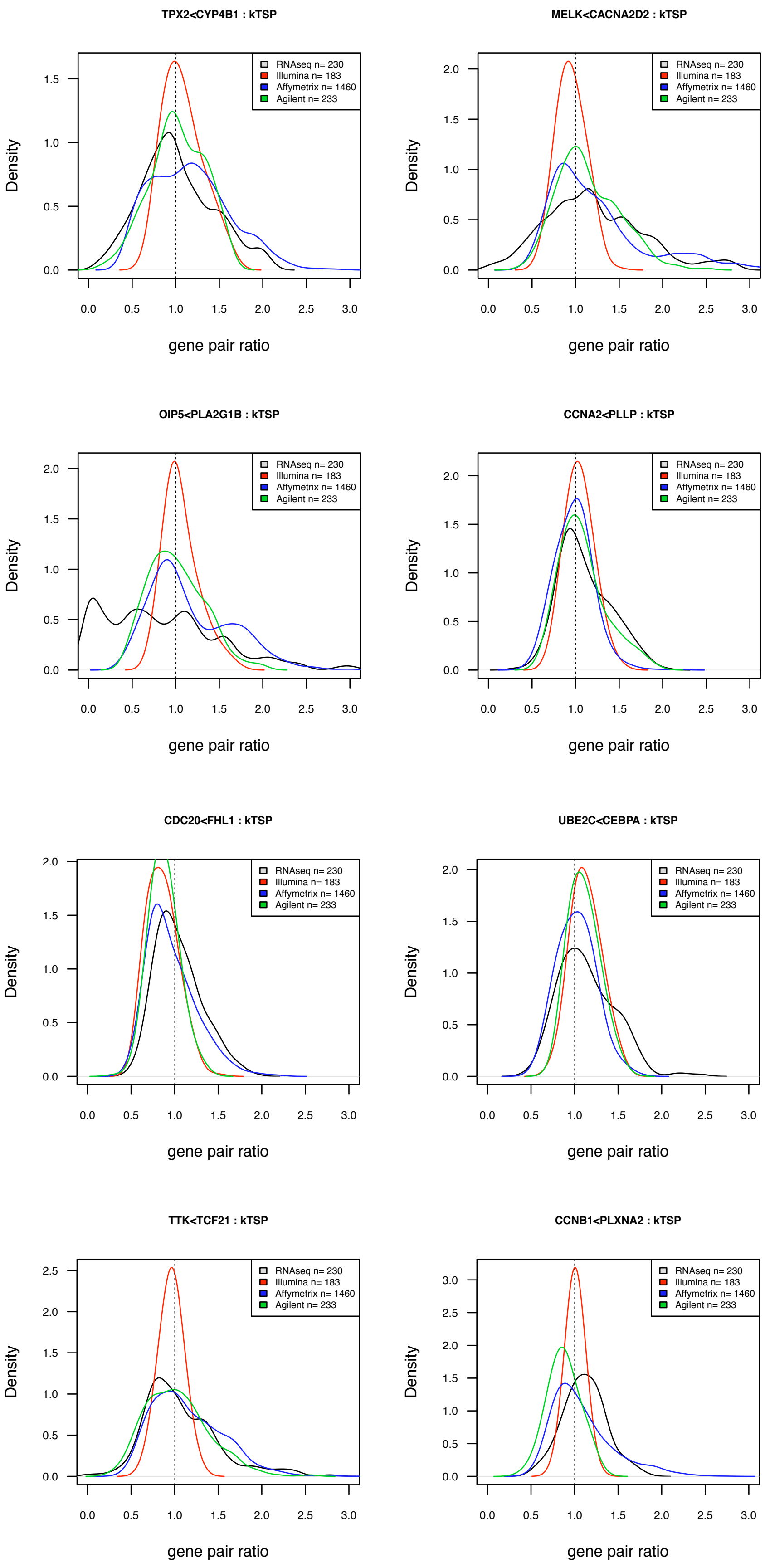
TPX2<CYP4B1 : kTSP
MELK<CACNA2D2 : kTSP
OIP5<PLA2G1B : kTSP
CCNA2<PLLP : kTSP
CDC20<FHL1 : kTSP
UBE2C<CEBPA : kTSP
TTK<TCF21 : kTSP
CCNB1<PLXNA2 : kTSP
RNAseq n= 230
Illumina n= 183
Affymetrix n= 1460
Agilent n= 233
Density
gene pair ratio

D)

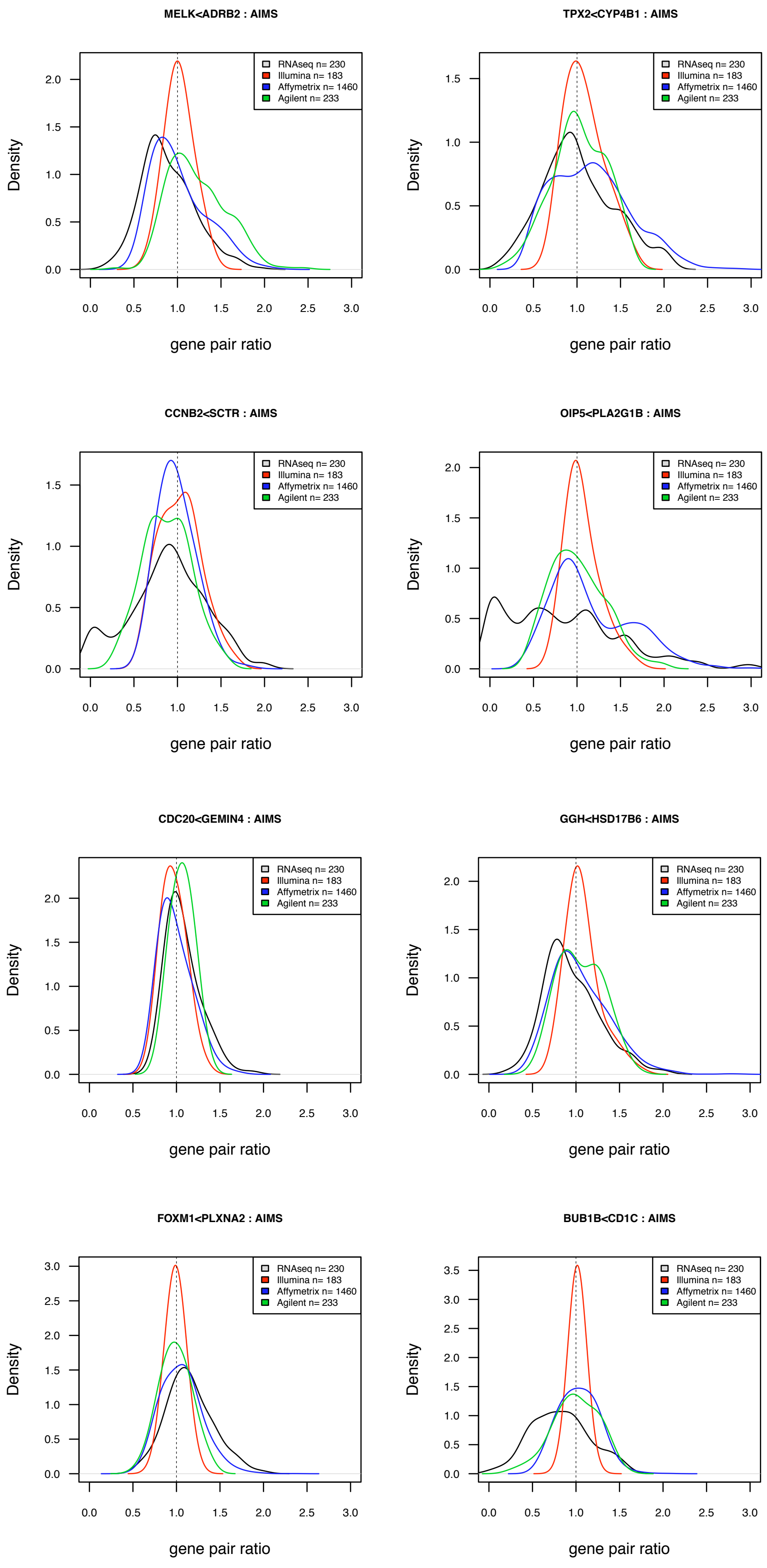
MELK<ADRB2 : AIMS
TPX2<CYP4B1 : AIMS
CCNB2<SCTR : AIMS
OIP5<PLA2G1B : AIMS
CDC20<GEMIN4 : AIMS
GGH<HSD17B6 : AIMS
FOXM1<PLXNA2 : AIMS
BUB1B<CD1C : AIMS
RNAseq n= 230
Illumina n= 183
Affymetrix n= 1460
Agilent n= 233
Density
gene pair ratio
0.0
0.5
1.0
1.5
2.0
2.5
3.0
3.5

SFigure 12.

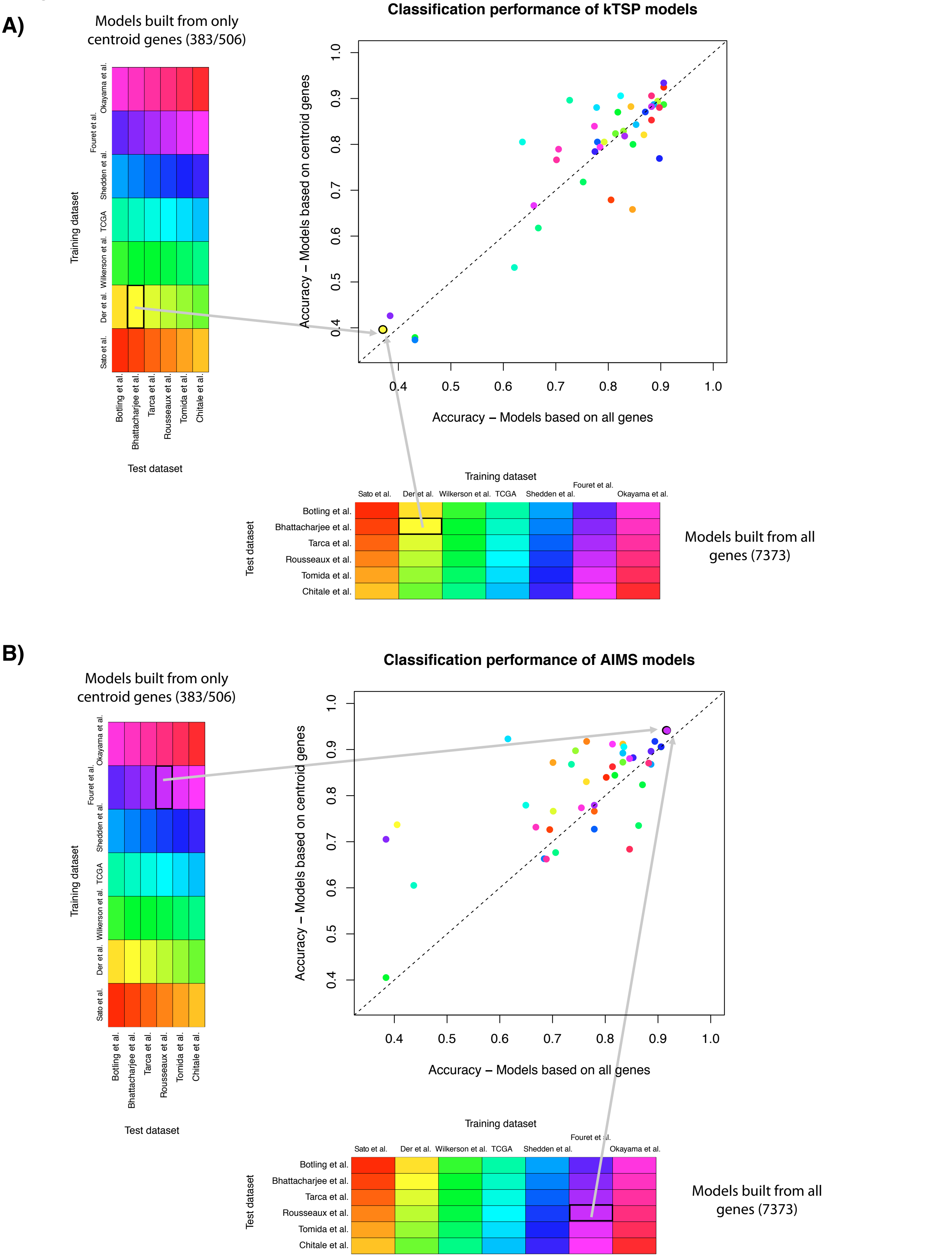

Supplementary Figure S12. Comparison of pre-feature selection for molecular subtype model development. (**A**) Comparison of performance in test sets for different kTSP models derived from training sets using either the full gene set (x-axis, corresponding to data in main manuscript) or genes available from the original subtype centroids only. (**B**) Similar analysis for the AIMS method.

SFigure 13.

A)

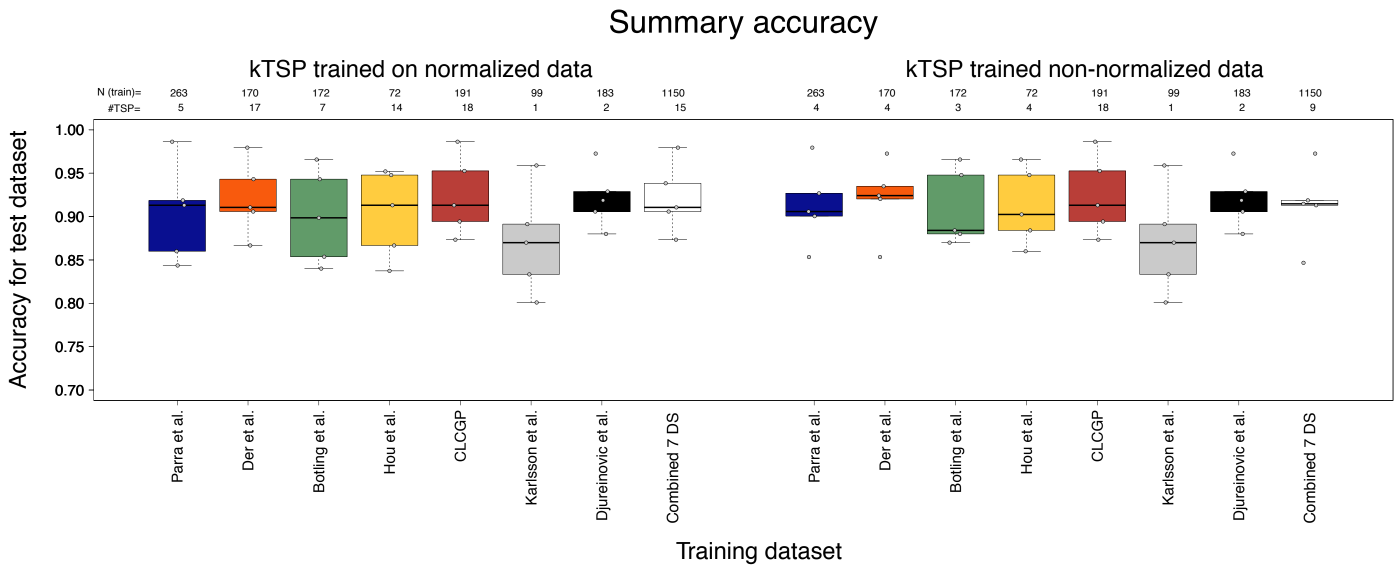

B)

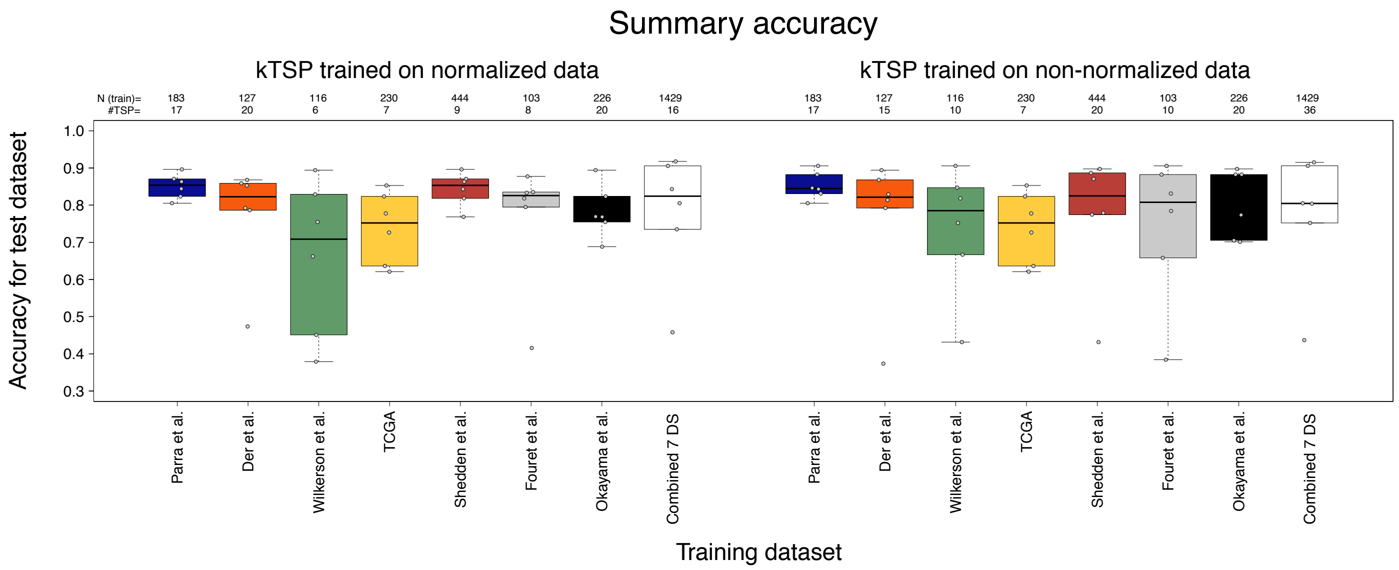

Supplementary Figure S13. Comparison of kTSP performance for non-normalized and normalized training data in the histological and molecular subtype arms. (**A**) Histological case study arm. Accuracy results in histologic test datasets for models trained in the seven individual training datasets plus a pooled training dataset of all individual datasets (7DS) and applied to the test datasets for kTSP using normalized training data (left) or non-normalized training data (right). All derived SSPs were applied to non-normalized test datasets. (**B**) Similar analysis for the molecular subtype case study arm.

SFigure 14.

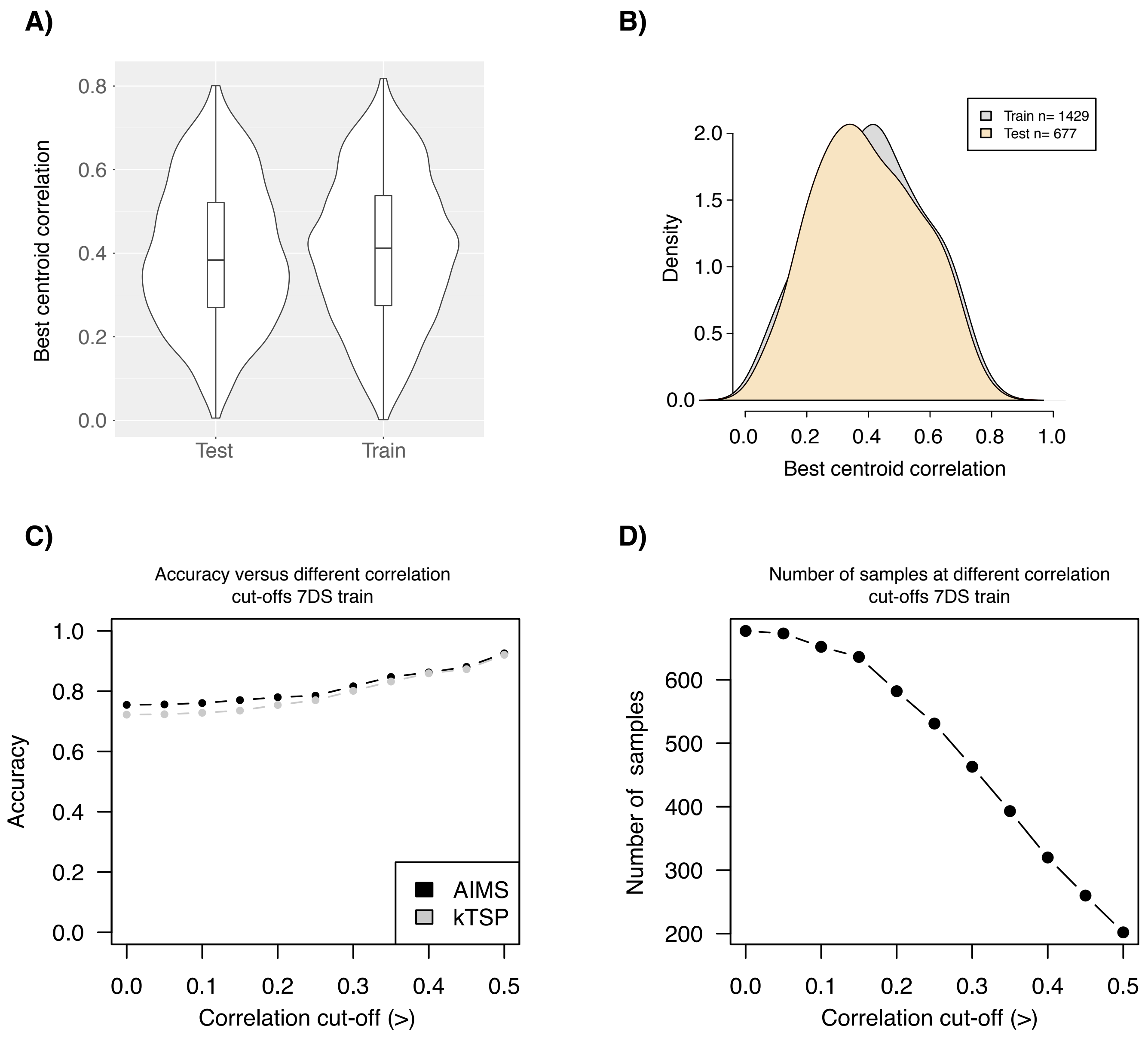

Supplementary Figure S14. Distribution of molecular subtype centroid correlation coefficients and influence on SSP prediction accuracy. (**A**) Violin plot with mean and IQR for the highest centroid correlation (i.e. the best correlation to a subtype centroid) for samples in the molecular subtype case arm divided by training and test data set assignment. (**B**) Corresponding kernel density plot of the centroid correlations stratified by training and test dataset assignment. (**C**) Prediction accuracy of AIMS and kTSP SSP models trained in the 7DS training dataset versus increasing correlation cut-off for all combined test dataset cases (i.e. patients from test datasets are pooled, n=677 patients in total). Only patients with a centroid correlation higher than a cut-off are used to calculate the accuracy, meaing different numbers for different cut-offs. (**D**) Corresponding number of samples used in C for increasing correlation cut-off. Numbers decrease with increasing correlation cut-off, meaning increasing numbers of unclassified samples (samples without a centroid class).
