# Supplementary material for: Performance of gene expression–based single sample predictors for assessment of clinicopathological subgroups and molecular subtypes in cancers: a case comparison study in non-small cell lung cancer
Source: Brief Bioinform. 2019 Feb 4;21(2):729–40. doi: 10.1093/bib/bbz008 (PMC7299291; doi:10.1093/bib/bbz008)
Supplement: Supp_bbz008 [file supp_bbz008.zip › Supplementary_Methods.pdf]

SUPPLEMENTARY MATERIAL AND METHODS

Performance of gene expression based single sample predictors for assessment of clinicopathological subgroups and molecular subtypes in cancers: a case comparison study in non-small cell lung cancer

Helena Cirenajwis[1], Martin Lauss[1], Maria Planck[1], Johan Vallon-Christersson[1], Johan Staaf[1]

[1] Division of Oncology and Pathology, Department of Clinical Sciences Lund, Lund University, Medicon Village, SE 22381 Lund, Sweden

Corresponding author: Johan Staaf, Associate Professor (johan.staaf@med.lu.se)

SUPPLEMENTARY MATERIAL AND METHODS

Raw data

Affymetrix

Raw data for Affymetrix datasets was extracted using the threestep() function in the Affy R-package using default parameters except for normalization=FALSE. To note, for GSE8894 we could not obtain strict raw data as CEL files for this dataset is not publicly available. In this case we used normalized data as surrogate for raw data.

Agilent

Raw channel data for Agilent data (dual-color arrays) was extracted directly from the scanner files for the designated sample channel (usually Ch2) based on the column "rProcessedSignal".

Illumina

Raw data from Illumina datasets were obtained from deposited data in Gene Expression Omnibus. To note, for CLCGP strict raw data is not available as the original authors have not made it publicly available. In this case we used the publicly available normalized data as surrogate.

RNAseq

Raw data for RNAseq datasets were obtained as deposited data from respective site (TCGA or Gene Expression Omnibus). For GSE81089 an offset of +1 was added prior to log2 transformation to avoid negative values.

Probe annotations were extracted either from deposited data, or through the gcrma function for Affymetrix data. All data was harmonized to include only RefSeq genes present on all platforms. In instances with duplicated probe identifiers, the most varying probe was kept in the respective dataset. Each gene was represented by the mean of multiple probes for that specific gene if multiple gene identifiers were present. The above procedure was performed strictly for creating a uniform, easy to use expression dataset in which raw data from all cohorts could be merged into one large matrix.

Normalized data

For Agilent data, normalized gene expression data was obtained from the respective Gene Expression Omnibus repository.

For Illumina data, normalized gene expression data was obtained from the respective Gene Expression Omnibus repository or from original authors web-site (CLCGP).

Affymetrix datasets were normalized using the gcrma() function from the gcrma R-package, with default settings. Each dataset was normalized individually.

Probe annotations were extracted as described above.

Merging individual datasets into large cohorts

For each case study arm, training datasets were concatenated on gene symbol without any batch adjustment. Individual datasets comprised non-normalized gene expression data, which had been preprocessed (one probe corresponding to one gene symbol).

Nearest centroid classification to assign molecular subtype (TRU / non-TRU)

In order to classify samples into the molecular subtypes TRU or non-TRU (comprising the groups PP and PI), datasets in the molecular case study arm were subjected to nearest centroid classification (NCC) using the 506-gene centroids as previously described [1]. Normalized and gene-centered (median) data were matched against the centroids on gene symbol (383 genes overlapped across all datasets for this case study arm). Matched centroid genes were extracted (multiple probes for each gene were averaged) and NCC was used to classify samples according to their maximum correlation (Pearson's correlation, no cut-off) of the sample with into any of the three centroids. A two-groups solution comprising TRU versus non-TRU cases was generated [2].

kTSP classifier

All functions described below were provided by the R package "switchBox" for building (training) and testing models developed by the kTSP algorithm [3]. If not stated otherwise,

default settings were used. Depending on the experimental case study arm, the kTSP classifier was trained against either the histopathology assessment (AC/SqCC) or the molecular subtypes (TRU/non-TRU) using the training function (SWAP.Train.KTSP). The input measurement was non-normalized gene expression data for each training dataset separately or a merged constellation of all training datasets (7DS). The candidate number of TSPs was set to 1-50, i.e. the number of TSPs that could be included in the model, whereas the optimal number of TSPs used in the final model was selected from the TSP scores from all the candidate pairs. Thus, for each experimental arm, eight kTSP models were developed. Each developed kTSP classifier was applied to the assigned test datasets (non-normalized data) using the classification function (SWAP.KTSP.Classify) for predicting the new samples into any of the output classes. By default, each sample was classified according to the class voted by the majority of the TSPs ("majority wins" principle) by the classifier. Classification performance was evaluated by comparing the reference classification with the predicted outcome to obtain accuracy and balanced accuracy metrics (SWAP.GetKTSP.Result).

AIMS classifier

R scripts and necessary functions for building and testing models developed by the AIMS algorithm was kindly provided by the creator behind the original AIMS package [4]. We investigated the AIMS approach using exactly the same study outline as described for the kTSP classifier (described above). In general, the AIMS classifier follows a similar procedure as the kTSP classifier to identify a predictive set of rules (TSP) capable of distinguishing between different output classes [4, 5]. To select the optimal number of rules to generate maximum statistics on the agreement between AIMS and the training dataset, we let AIMS perform a 5-fold cross-validation on the training dataset and for each n-partition it was trying out a range of selected rules (1 to 50) to create 50 distinct Naïve Bayes classifiers, which were validated on the test subset within each of the cross-validation partitions. This was repeated 10 times to achieve the final model based on the summarized results. To avoid unequal rule contribution in rule selection due to size differences across datasets for the merged training dataset, the AIMS algorithm applied a weighted form of rule selection provided by the R package "Rgtsp". In contrast to the kTSP classifier integrating the resulting rules using the majority voting scheme, AIMS uses a single Naïve Bayes classifier providing the posterior

probabilities for each output class. The Naïve Bayes classifier was called from the R package "e1071".

References

1. Wilkerson MD, Yin X, Walter V et al. Differential pathogenesis of lung adenocarcinoma subtypes involving sequence mutations, copy number, chromosomal instability, and methylation, PLoS ONE 2012;7:e36530.
2. Ringner M, Jonsson G, Staaf J. Prognostic and Chemotherapy Predictive Value of Gene-Expression Phenotypes in Primary Lung Adenocarcinoma, Clin Cancer Res 2016;22:218-229.
3. Afsari B, Fertig EJ, Geman D et al. switchBox: an R package for k-Top Scoring Pairs classifier development, Bioinformatics 2015;31:273-274.
4. Paquet ER, Hallett MT. Absolute assignment of breast cancer intrinsic molecular subtype, J Natl Cancer Inst 2015;107:357.
5. Tan AC, Naiman DQ, Xu L et al. Simple decision rules for classifying human cancers from gene expression profiles, Bioinformatics 2005;21:3896-3904.
